# Vesalius: high‐resolution *in silico* anatomization of spatial transcriptomic data using image analysis

**DOI:** 10.15252/msb.202211080

**Published:** 2022-09-06

**Authors:** Patrick C N Martin, Hyobin Kim, Cecilia Lövkvist, Byung‐Woo Hong, Kyoung Jae Won

**Affiliations:** ^1^ Department of Computational Biomedicine Cedars‐Sinai Medical Center Hollywood CA USA; ^2^ Biotech Research and Innovation Centre (BRIC) University of Copenhagen Copenhagen Denmark; ^3^ Computer Science Department Chung‐Ang University Seoul Korea

**Keywords:** anatomical territories, spatial domains, spatial transcriptomics, tissue architecture, tissue heterogeneity, Computational Biology, Methods & Resources

## Abstract

Characterization of tissue architecture promises to deliver insights into development, cell communication, and disease. *In silico* spatial domain retrieval methods have been developed for spatial transcriptomics (ST) data assuming transcriptional similarity of neighboring barcodes. However, domain retrieval approaches with this assumption cannot work in complex tissues composed of multiple cell types. This task becomes especially challenging in cellular resolution ST methods. We developed Vesalius to decipher tissue anatomy from ST data by applying image processing technology. Vesalius uniquely detected territories composed of multiple cell types and successfully recovered tissue structures in high‐resolution ST data including in mouse brain, embryo, liver, and colon. Utilizing this tissue architecture, Vesalius identified tissue morphology‐specific gene expression and regional specific gene expression changes for astrocytes, interneuron, oligodendrocytes, and entorhinal cells in the mouse brain.

## Graphical abstract

Vesalius is a computational tool for recovering tissue territories from spatial transcriptomics data using image processing technology. Vesalius illustrates how uniform tissue territories can help understanding tissue heterogeneity.

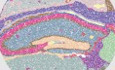

## Introduction

From the smallest cell to the largest organ, we find patterns of organization supporting homeostasis within organisms. Each cell functions in the context of its neighbors, and each group of structured cells functions in the context of tissue architecture. The investigation of this multi‐level organization promises to deliver insight into development, cellular communication, and disease.

One approach to probe into the organization of tissues and cells is by using spatial transcriptomics (ST) (Burgess, 2019; Rao *et al*, 2021). ST is a set of methods that recover gene expression while maintaining the spatial component intact. Several ST methods have been developed in the last few years and fall into two categories: image‐based approaches using fluorescence *in situ* hybridization (FISH) and sequencing‐based approaches using spatially resolved barcodes. Image‐based ST approaches including seqFISH (Lubeck *et al*, 2014; Eng *et al*, 2019; Lohoff *et al*, 2021) or merFISH (Chen *et al*, 2015) provide sub‐cellular resolution ST. Image‐based techniques rely on the pre‐selection of target mRNA species and—due to the challenge of distinguishing overlapping fluorescent signals—are limited to a smaller number of genes sampled at a time (Zhuang, 2021).

In contrast to image‐based ST, sequencing‐based ST techniques such as 10X Visium (Ståhl *et al*, 2016), Seq‐Scope (Cho *et al*, 2021), or Slide‐seq (Rodriques *et al*, 2019; Stickels *et al*, 2021) provide a non‐biased and genome‐wide quantification of mRNA species. Technological advances have made it possible to obtain sequencing‐based ST data on cellular and even sub‐cellular resolutions. Tissue territory detection in high‐resolution ST data will strengthen our understanding of tissue architecture and its associated marker genes providing further opportunities to study transcriptomic changes due to local environment and tissue morphology.

Several tools including Seurat (Satija *et al*, 2015), BayesSpace (Zhao *et al*, 2021), STAGATE (Dong & Zhang, 2022), SpaGCN (Hu *et al*, 2021), SEDR (preprint: Fu *et al*, 2021a), and Giotto (Dries *et al*, 2021) have been developed to understand tissue architecture from ST data. Seurat leverages reference single‐cell data sets to map barcode identities to their respective location in ST data. While this approach demonstrates the cellular heterogeneity of tissues, the task of recovering and extracting anatomical regions is still challenging due to their cellular complexity. On the contrary, BayesSpace and Giotto provide distinct models both utilizing Hidden Markov random fields. Their respective methods attempt to cluster barcodes together under the assumption that neighboring barcodes are likely part of the same cluster if transcriptionally similar. These methods have performed well in low‐resolution Visium 10X data (Dries *et al*, 2021; Zhao *et al*, 2021). However, this assumption will not necessarily hold in complex tissue containing multiple cell types as neighboring barcodes are just as likely to represent a different cell type in high‐resolution ST data. Approach using auto‐encoders and adaptive graph attention auto‐encoders can generate a spatially aware latent space upon which clustering methods are applied. This has been achieved by (preprint: Fu *et al*, 2021a; Dong & Zhang, 2022). The recovery of anatomical structures containing multiple cell types is extremely arduous and has relied on manual isolation. One solution to this challenge is to link anatomical territories from companion hematoxylin and eosin (H&E) staining images to their spatial transcriptomic assay (Bergenstråhle *et al*, 2020; preprint: Pham *et al*, 2020; Hu *et al*, 2021; preprint: Peng *et al*, 2021; Palla *et al*, 2022). However, techniques such as Slide‐Seq (Rodriques *et al*, 2019; Stickels *et al*, 2021) do not provide these companion images. Furthermore, the segmentation of anatomical territories in images remains challenging without manual annotation (Gurcan *et al*, 2009; Vu *et al*, 2019; Kurc *et al*, 2020).

To address these limitations, we developed Vesalius—an R package—designed to perform *in silico* anatomization and isolation of tissue territories without the use of H&E companion images. Vesalius converts the transcriptome into an RGB color code that is then embedded into an image. By leveraging a variety of image analysis techniques, Vesalius can recover complex tissue territories in the mouse brain, mouse embryo, diseased liver, and colon. Comprehensive tests using simulated data indicate that Vesalius can identify tissue territories even when the tissue is composed of multiple cell types while other competitors often identify numerous unwanted patches. Identified territories are used to discover tissue morphology‐specific gene expression as well as changes in the transcriptome of astrocytes, interneuron, oligodendrocytes, and entorhinal cells in the mouse brain depending on the tissue territory.

## Results

### Vesalius embeds the transcriptome in the RGB color space

**Figure 1:**
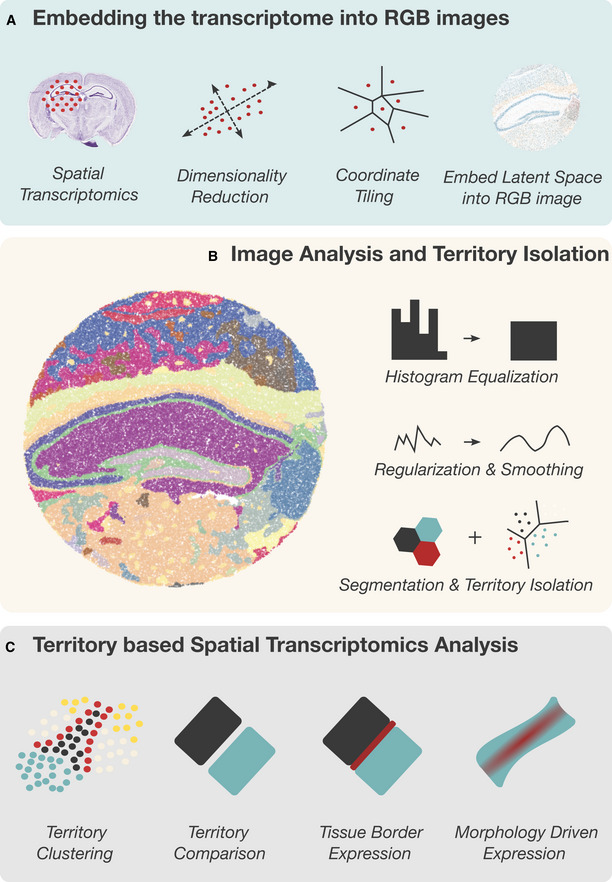
Describing Tissue territories with Vesalius AVesalius embeds ST data into RGB‐colored images. This is achieved by preprocessing ST data and reducing dimensionality. In parallel, punctual ST coordinates are converted into tiles. Finally, the UMAP latent space (or PCA loading values) is transformed into an RGB color space, and the color code attributed to each barcode is assigned to its respective tile.BVesalius applies image analysis techniques to RGB images describing the transcriptional landscape of a tissue with the aim of isolating tissue territories.CVesalius enables a territory‐based ST framework including spatial territory clustering, territory comparison, tissue border expression, and morphology‐driven expression.

**Figure EV1:**
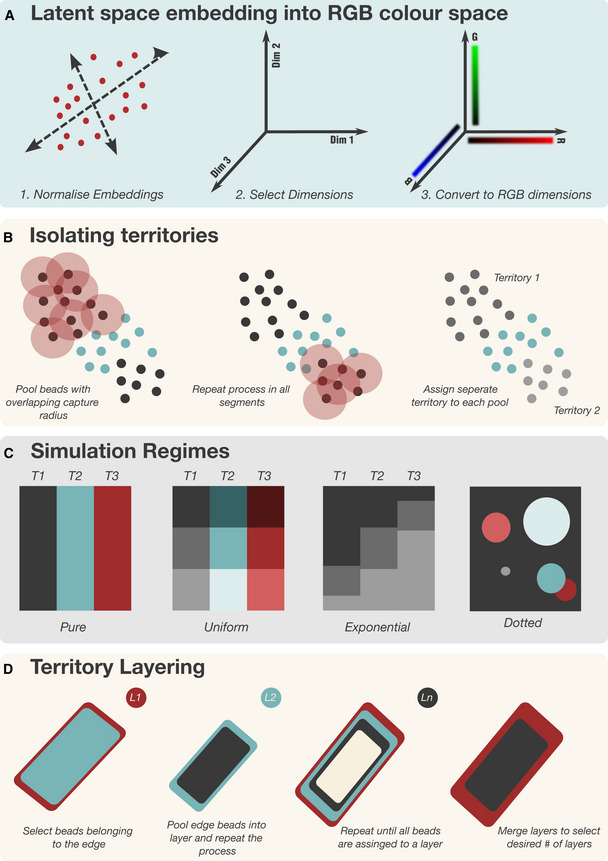
Vesalius methods visualized AVesalius converts the transcriptome of cells by using a normalized latent space (either UMAP or PCA) and using embedding values as RGB color values.BAfter image segmentation, Vesalius further isolates territories by pooling barcodes that are close to each other in 2D space. Vesalius finds all barcodes that are within a capture distance of each other (represented by red circle) and assigns a unique territory to all these barcodes. The same process is applied to all beads of that color segment until all beads have been pooled into a distinct spatial territory.CSimulation regimes used to benchmark Vesalius in high‐resolution ST data. The pure regime only contains one cell type per territory. The uniform regime contains n different cell types in equal proportion in each territory. The exponential regime contains n cell types in varying proportions between territories. The cell types are the same between territories. The dotted regime is made of a background territory with five circular territories of random size. The number of cell types between each territory is also randomized. Circular territories may overlap.DTerritory layering uses images representation of territory to iteratively select the edge of a territory and assign a layer value to that edge. Once all barcodes have been assigned to a layer, the number of layers can be reduced by merging neighboring layers.

The core concept of the Vesalius algorithm is to represent the transcriptome of a barcode as a color in the RGB color space and build images upon which image analysis techniques can be applied (Fig 1A and B). To embed the transcriptome into images, Vesalius first preprocesses sequencing‐based spatial transcriptomic data by log normalizing and scaling counts values and extracting highly variable features and reduces dimensionality via principal component analysis (PCA). Next, Vesalius uses Uniform Manifold Approximation and Projection (UMAP) to project PCs into three dimensions and embed the latent space into the RGB color space (see Materials and Methods—Fig EV1A). Alternatively, Vesalius can also embed PC loading values into the RGB color space for a more targeted view of data variance (see methods—Appendix Fig S1). Vesalius handles the uneven location of barcodes in the ST assay by expanding punctual coordinates into multi‐pixel tiles using Voronoi Tessellation. Images are constructed by associating color codes to their respective tile.

Next, Vesalius applies image analysis techniques to extract tissue territories (Fig 1B; Appendix Fig S2). After balancing the color histogram and smoothing (see Materials and Methods), image segmentation based on k‐means clustering is applied to produce color clusters that can be further subdivided into territories. Image processing parameters can be finely tuned depending on the user's interest and the ST method used. Vesalius checks if barcodes are within a certain capture radius of each other. Vesalius will assign barcodes belonging to the same color cluster into separate territories if they are far enough from each other in 2D space (see Materials and Methods—Fig EV1B).

The isolation of anatomical territories and image representation of ST data with Vesalius enhances ST analysis by providing a territory‐based framework (Fig 1C). Isolated territories can be further clustered to recover the finer details of cellular organization. Territories can be compared to investigate territory‐specific gene expression. Neighboring territories can be manipulated to recover tissue border gene expression and gene expression patterns arising within specific anatomical structures.

### Vesalius outperforms other spatial domain tools in heterogenous territories

To assess the performance of Vesalius, we simulated ST data under four different regimes: pure, exponential, uniform, and dotted (Fig EV1C). We compared the performance of Vesalius against Seurat (Satija *et al*, 2015), BayesSpace (Zhao *et al*, 2021), Giotto (Dries *et al*, 2021), SpaGCN (Hu *et al*, 2021), SEDR (preprint: Fu *et al*, 2021a), and STAGATE (Dong & Zhang, 2022) in these simulated data sets. The pure regime contains a single cell type in each territory. The uniform regime contains n different cell types in each territory, and each cell type appears in equal proportion. The exponential regime contains n cell types, and the overall proportion of each cell type changes between territories following an exponential pattern. For uniform and exponential regime, we evaluated the performance of each tool with *n* = 3, 4, and 5 cell types in each territory. Finally, the dotted regime consists of a background tissue territory in which five circular territories are placed. Both the background and circular territories contain a random number of cell types (between 1 and 3). In all regimes, simulated data sets contain 6,000 “cells” divided between territories (see Materials and Methods—Dataset EV1).

**Figure 2:**
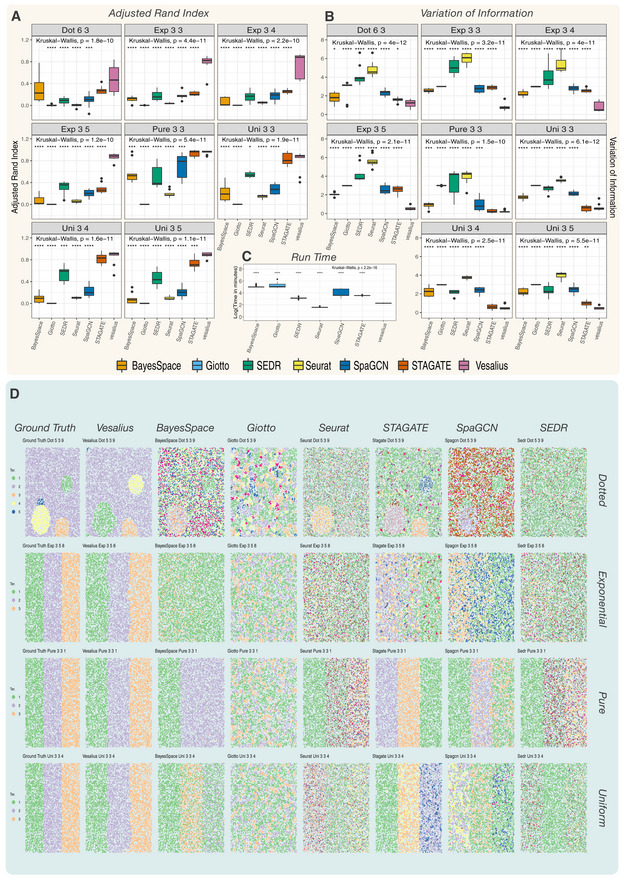
Vesalius outperforms other Spatial domain tools in heterogenous territories AAdjusted Rand Index score between Vesalius, BayesSpace, Giotto, Seurat, STAGATE, SpaGCN, and SEDR. Overall, Vesalius outperforms other tools in retrieving spatial domains in high‐resolution simulated data sets (Kruskal–Wallis and star notation using Wilcoxon rank‐sum test for the multiple comparisons with *n* = 10 simulation replicates).BThe high performance of Vesalius compared with competitors is also highlighted using the variation of information score, which is more robust against cluster granularity. Boxplot shows VI scores over 10 simulation replicates (Kruskal–Wallis and star notation using Wilcoxon rank‐sum test for the multiple comparisons with *n* = 10 simulation replicates).CVesalius has the lowest run time among spatial domain tools with a run time 3–20 times lower than competing tools (Kruskal–Wallis and star notation using Wilcoxon rank‐sum test for the multiple comparisons with *n* = 80 simulation replicates).DExample of simulated ground truth and prediction by each tool in four simulated regimes (Dotted, Exponential, Pure, and Uniform). The label above each plot describes the simulation run in the following format Tool–Regime–Number of territories–Number of cells–Simulation replicate. For example, Vesalius Uni 3 3 1 describes Vesalius's prediction in the uniform regime, which contains three territories each with three cell types in replicate 1. Data information: Box plots show five summary statistics: the median, two hinges representing the interquartile range (IQR) and two whiskers representing 1.5 times the IQR from the median. All dots are considered as outliers from this distribution.

To quantitatively assess the performance of Vesalius compared with other tools, we used an Adjusted Rand Index (ARI) (Rand, 1971). While ARI scores are often used to compare clustering performance, they may be affected by cluster granularity and as such we also compared the performance of each tool using a variation of information (VI) metric (Meilǎ, 2007). Overall, Vesalius outperformed competing tools in all regimes (Kruskal–Wallis and Wilcoxon rank‐sum test for multiple comparisons with *n* = 10 simulation replicates per regime—*P*‐values shown in Fig 2A and B) in both ARI scores and VI scores (Fig 2A and B). Only STAGATE provided a similar ability to recover tissue territories in subset of simulation regimes when comparing both ARI and VI scores. Additionally, Vesalius has the lowest run time among spatial domain tools with a run time 3 to 20 times lower than competing tools (Kruskal–Wallis and Wilcoxon rank‐sum test for multiple comparisons with *n* = 80 simulation replicates over all regimes—*P*‐value < 2.2e‐16 shown in Fig 2C).

In these simulations, Vesalius successfully recovered territories across all conditions (Fig 2D). Vesalius can clearly distinguish territories containing single cell types (pure), different cell types (uniform) or differences in cell‐type proportions (exponential). In contrast to other tools that drastically overestimate the number of tissue patches, Vesalius merges smaller territories spots into larger neighboring ones (dotted). However, this loss of smaller territories can easily be recovered by downstream analysis of isolated territories. BayesSpace, SpaGCN, SEDR, and STAGATE partially recovered territories in the uniform, pure, and dotted regimes. In all cases, these tools overestimate the number of true territories and struggle to clearly distinguish territories that contain multiple cell types or cell types in varying proportions. Seurat did not clearly recover tissue territories; however, this is unsurprising since Seurat was not designed to recover tissue territories. It is remarkable that Giotto did not recover any territory even in the pure regime and only identified numerous small patches.

### Vesalius overcomes the challenge of isolating tissue territories containing heterogenous cell populations

High‐resolution sequencing‐based ST methods promise an unbiased and spatially resolved view of the transcriptome. Yet, most tissues contain multiple cell types, and it can be challenging to recover uniform anatomical structures especially when no companion H&E images are provided. To demonstrate Vesalius's ability to isolate tissue territories, we used Slide‐seq V2 that provides a high‐resolution sequencing‐based ST assay for mouse hippocampus and embryo (Data ref: Stickels *et al*, 2021).

**Figure 3:**
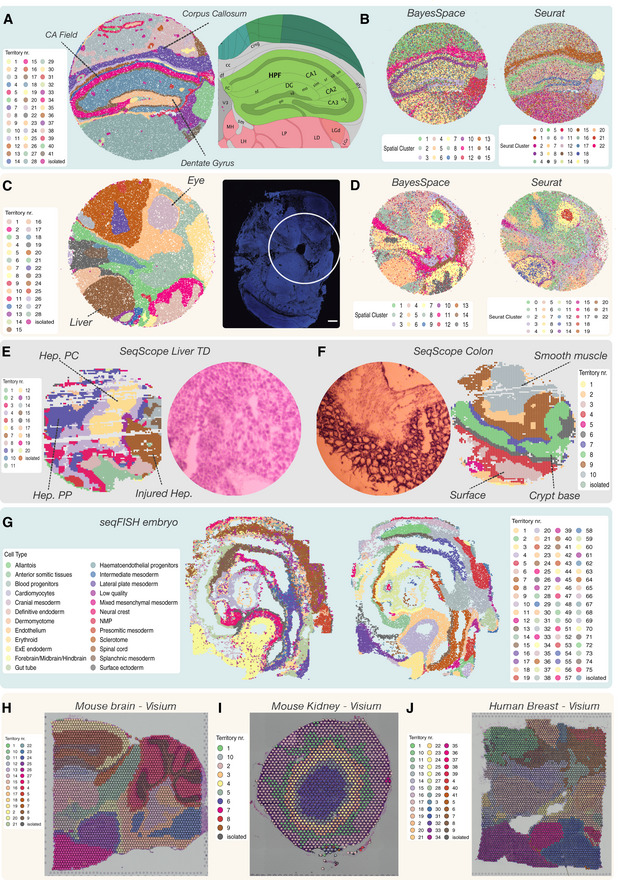
Vesalius recovers uniform anatomical territories in high‐resolution ST data AVesalius accurately recovers tissue territories in Slide‐seqV2 data taken from the mouse hippocampus and surrounding brain (Puck_200115_08). A comparison with the Allen Brain Atlas reference atlas illustrates that Vesalius recovers many structures such as the dentate gyrus, corpus callosum, and the CA field.BBayesSpace and Seurat applied to the same data set recover structures insofar as these structures contain homogenous cell populations. The identified clusters are dispersed over the entire tissue section and thus do not represent a clear tissue territory.CVesalius recovered uniform tissue territories in the mouse embryo (Slide‐seqV2—Puck_190926_03). The microscopy image highlights the section of the embryo used to produce Puck_190926_03 (image taken from Slide‐seq V2; Stickels *et al*, 2021).DBayesSpace and Seurat recover structures insofar as these structures contain homogenous cell populations. The identified clusters are dispersed over the entire tissue section and thus do not represent a clear tissue territory.EVesalius identified tissue territory using Seq‐Scope early‐onset liver failure (Sample 2117). Vesalius highlights various hepatocyte populations such as pericentral hepatocytes (Hep. PC), periportal hepatocytes (Hep. PP), and injured hepatocytes (Injured Hep.).FVesalius identified tissue territory using Seq‐Scope in healthy colon (Sample 2111). Territory isolation in the colon shows various structures and layers including the smooth muscle, the crypt surface, and the crypt base.GVesalius recovers tissue territories in seqFISH data in mouse embryo (right) and can distinguish between different brain regions compared with the single‐cell annotation (left).H–JIn low‐resolution Visium 10X data sets, Vesalius accurately recovers tissue territories in a broad range of tissue such as mouse brain (H), mouse kidney (I), and human breast cancer (J).

**Figure EV2:**
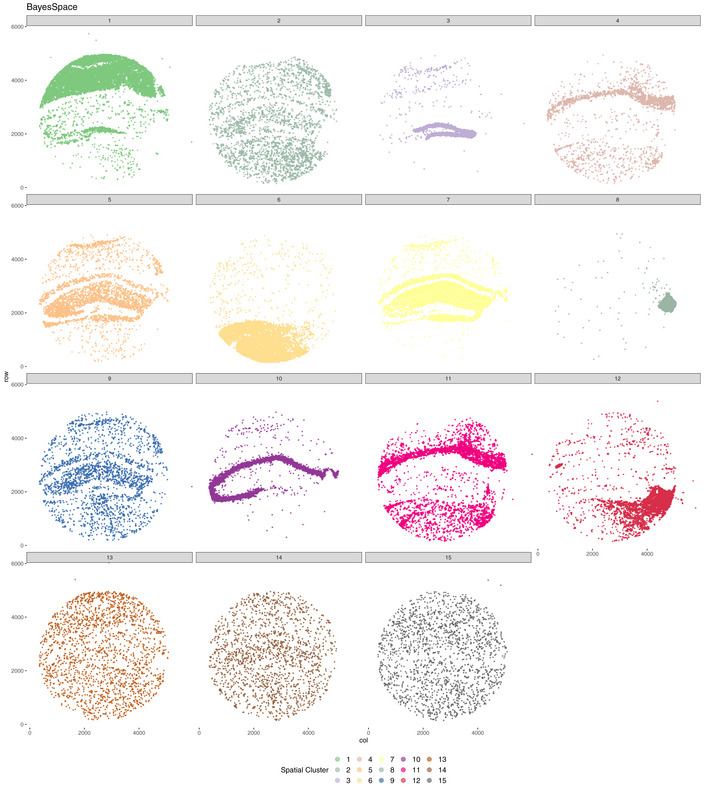
BayesSpace spatial cluster in mouse hippocampus Visualizing BayesSpace clusters separately emphasizes that many of the spatial domains do not represent a specific anatomical structure. In this context, selecting a specific location in the tissue is impossible without either missing barcodes or by including unwanted barcodes. This also demonstrates why cluster sub‐clustering does not equate to territory isolation and clustering.

**Figure EV3:**
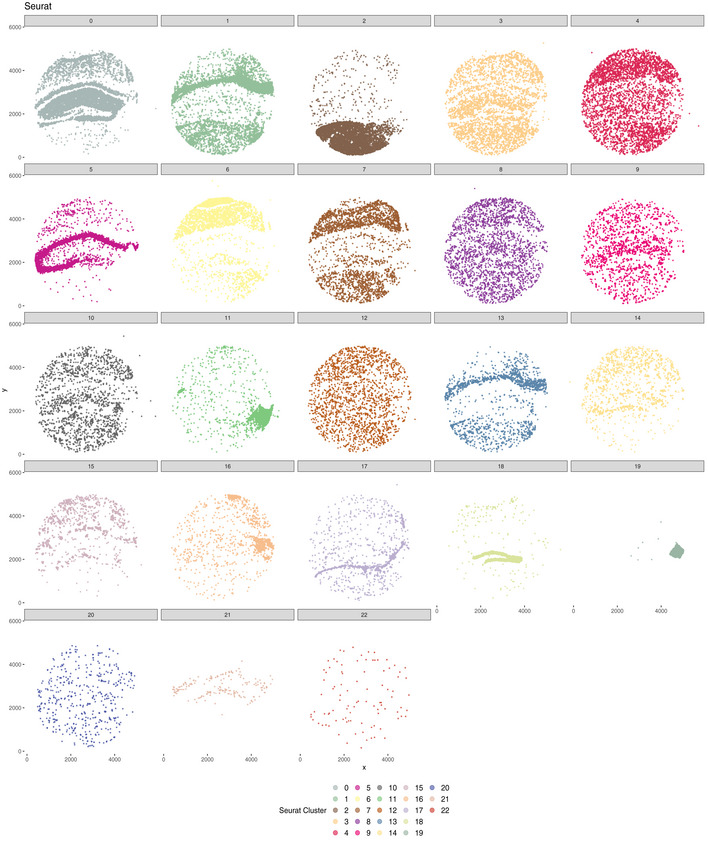
Seurat clusters in mouse hippocampus Visualizing Seurat clusters separately emphasizes that many of the clusters do not represent a specific anatomical structure. In this context, selecting a specific location in the tissue is impossible without either missing barcodes or by including unwanted barcodes. This also demonstrates why cluster sub‐clustering does not equate to territory isolation and clustering. Seurat is not designed to recover spatial domains but rather cluster transcriptional similarity.

Vesalius identified 41 territories in the mouse hippocampus (Puck_200115_08) including the CA field, the dentate gyrus, and the corpus callosum (Fig 3A—Image is from Allen Institute; Data ref: Lein *et al*, 2007). Territories characterized by too little barcodes (< 50) and too far away from another territory were described as isolated (see Materials and Methods). Territories recovered by Vesalius are characterized by uniform regions that match well with reference annotation (Fig 3A). In contrast to Vesalius, BayesSpace (Zhao *et al*, 2021) and Seurat (Satija *et al*, 2015) recover tissue territories insofar as these structures are characterized by a homogenous population of cells but produce numerous small patches potentially due to the heterogenous nature of the brain tissues (Figs 3B and EV2, and EV3).

**Figure EV4:**
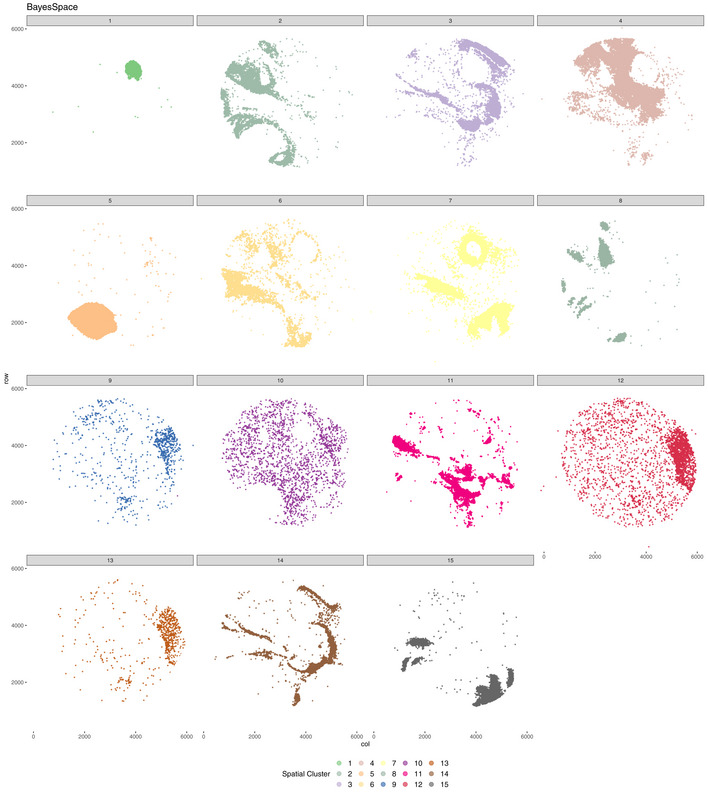
BayesSpace spatial cluster in mouse embryo Visualizing BayesSpace clusters separately emphasizes that many of the spatial domains do not represent a specific anatomical structure. In this context, selecting a specific location in the tissue is impossible without either missing barcodes or by including unwanted barcodes. This also demonstrates why cluster sub‐clustering does not equate to territory isolation and clustering.

**Figure EV5:**
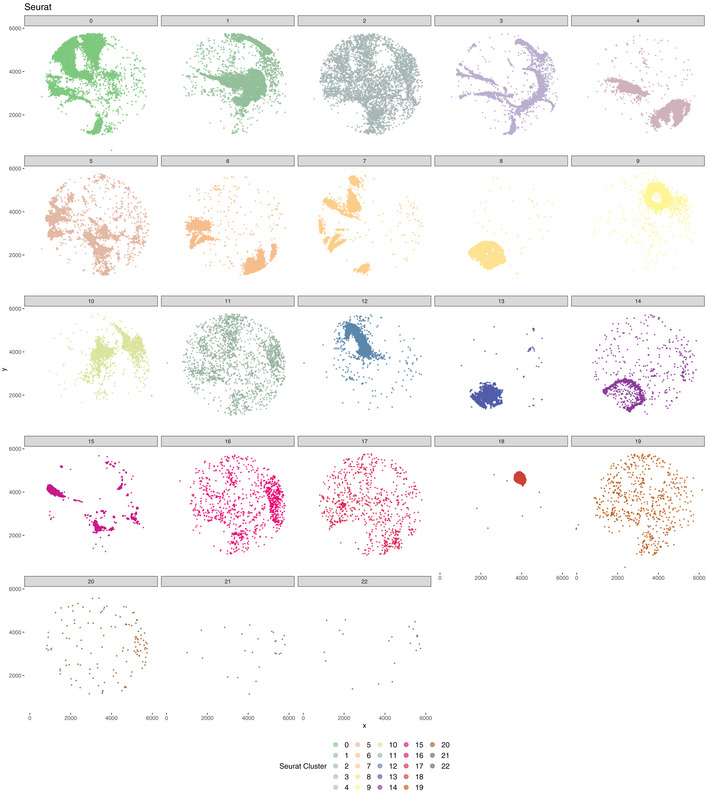
Seurat clusters in mouse embryo Visualizing Seurat clusters separately emphasizes that many of the clusters do not represent a specific anatomical structure. In this context, selecting a specific location in the tissue is impossible without either missing barcodes or by including unwanted barcodes. This also demonstrates why cluster sub‐clustering does not equate to territory isolation and clustering. Seurat is not designed to recover spatial domains but rather cluster transcriptional similarity.

We observed a similar phenomenon when we analyzed the mouse embryo Slide‐seqV2 data set (Puck_190926_03). Vesalius was able to recover 28 uniform territories such as the embryonic liver and eye (Fig 3C). As expected, it is not easy to define clear territories with BayesSpace and Seurat as numerous small regions were identified (Figs 3D and EV4, and EV5).

### Vesalius illustrates tissue territories in a broad range of ST data sets

The challenge of heterogeneity is further amplified in sub‐cellular resolution ST data as the transcriptome of each cell will likely be spread between multiple barcodes. Seq‐Scope (Date ref: Cho *et al*, 2021) is an ST assay with a resolution smaller than 1um. To test whether we can still recover territories, we applied Vesalius to murine Seq‐Scope (Data ref: Cho *et al*, 2021) data in early‐onset liver failure and crypt‐surface colon (Fig 3E and F). Vesalius illustrates hepatocellular zonation with both pericentral hepatocytes (Hep. PC) and periportal hepatocytes (Hep. PP) (Fig 3E). We were also able to recover a territory of injured hepatocytes (Injured Hep.). Deciphering of tissue structure proved to also be successful in the colon where Vesalius shows territories related to smooth muscle, the crypt base, and the crypt surface (Fig 3F). Interestingly, our results suggest the emergence of different layers within the smooth muscle and crypt base.

Next, we challenged Vesalius to recover tissue territories when a smaller number of genes are being probed simultaneously. We used seqFISH data taken from embryo slices (Data ref: Lohoff *et al*, 2021) and demonstrate that Vesalius can recover tissue territories within image‐based ST data (Fig 3G). We were able to summarize tissue territories including different regions of the brain such as the forebrain, midbrain, and hindbrain. Interestingly, our results suggest that this distinction might be overly simplistic as we observed a supplementary layer at the interface between the brain and the cranial mesenchyme.

Finally, we investigated whether Vesalius could recover tissue territories in lower‐resolution ST such as it is the case in Visium 10X. Vesalius was able to accurately recover tissue territories in broad range of samples in both mouse brain and kidney (Fig 3H and I) but also human breast cancer (Fig 3J). Our results suggest a ringed structure surrounding the kidney medulla.

### Isolating territories highlights the finer details of spatial patterning

We have shown that Vesalius is able to recover and isolate tissue territories from high‐resolution ST data, especially tissue territories containing heterogenous cell populations. The isolation of territories enables an in‐depth view of spatial patterning uncovering subtle expression patterns, new anatomical compartments, and spatially resolved transcriptional shifts.

**Figure 4:**
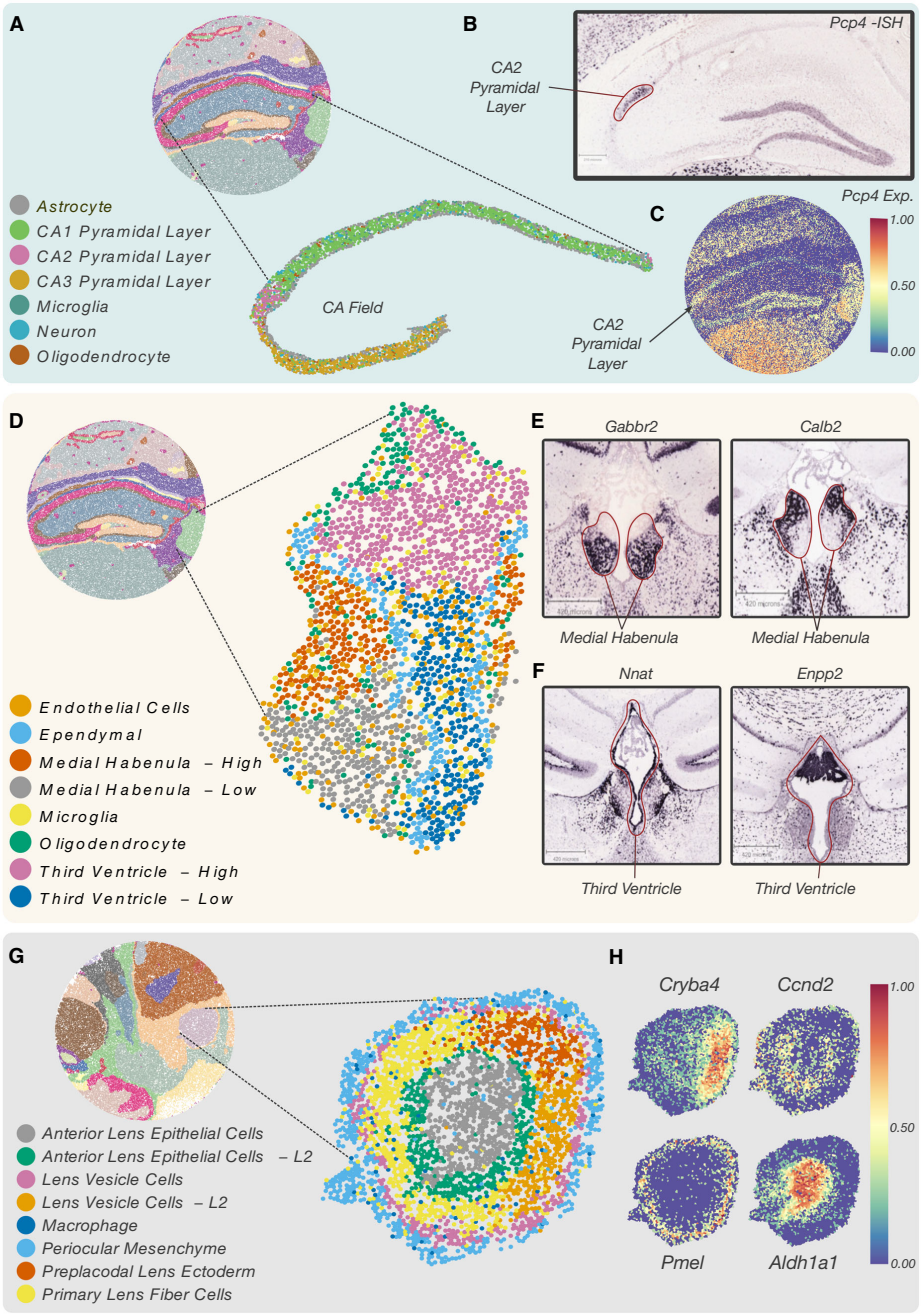
In‐depth analysis of isolated territories reveals the finer details of spatial patterning AMapping of clustered barcodes in the isolated CA field. Vesalius recovers all 3 CA pyramidal layers.BCA2 pyramidal layer was enriched with, among others, *Pcp4*, a canonical CA2 layer marker. The ISH image taken from the Allen Brain Atlas corroborates the positioning of the CA2 layer within the isolated CA field.CPcp4 expression within the CA2 layer is lost in favor of stronger expression in the thalamus.DThe isolated medial habenula and third ventricle show distinct spatial compartments after barcode clustering.EISH image describing a medial habenula lower compartment marker (*Gabbr2*) and a medial habenula upper compartment marker (*Calb2*). The medial habenula is highlighted in red.FISH images of lower third ventricle marker (*Nnat*) and upper third ventricle marker (*Enpp2*).GBarcode clustering and mapping of the embryonic eye (E12.5) show that Anterior Lens Epithelial cells and Lens Vesicle cells are separated into distinct layers.HDifferential gene expression analysis between Anterior Lens Epithelial Cell layers revealed that the expression of *Cryba4* was restricted to the inner layer while *Cnnd2* was expressed in the outer layer. Similarly, *Pmel* was expressed in the outer Lens Vesicle cell layer and *Aldh1a1* was expressed in the inner layer.

For instance, the analysis of the isolated CA field recovers all three CA field cell types namely CA1 pyramidal cells, CA2 pyramidal cells, and CA3 pyramidal cells (Fig 4A). In contrast, BayesSpace and Seurat were unable to recover all three sections (Fig 3B). We identified *Pcp4*, *Rgs14*, and *Necab2* as marker genes for CA2, which is consistent with recent proteomics study for CA2 against CA1 (Gerber *et al*, 2019). The *in situ* hybridization (ISH) images against these genes taken from the Allen Brain Atlas (Lein *et al*, 2007) validate our prediction (Fig 4B, Appendix Fig S3). Further investigation of *Pcp4* expression illustrates that this gene has a strong expression in the thalamus and a comparatively weak expression in the CA2 field (Fig 4C) and territory isolation by Vesalius enabled detection of the CA2 field in this noisy data.

The isolation of the third ventricle and the medial habenula further demonstrates how subtle gene expression patterns can be retrieved by using Vesalius. Indeed, the medial habenula and the third ventricle are divided into distinct spatial compartments (Fig 4D). We observed an upper and lower medial habenula compartment characterized by distinct gene expression patterns. Overall, 119 genes were differentially expressed (*P* < 0.05—Wilcoxon rank‐sum test) between both compartments (Dataset EV3 and EV4). For example, *Gabbr2* showed a higher expression in the lower medial habenula compartment (Fig 4E—left) while *Calb2* showed a higher expression in the upper compartment (Fig 4E—right). The medial habenula is highlighted in red. Similarly, the third ventricle exhibited 288 differentially expressed (*P* < 0.05—Wilcoxon rank‐sum test) between the upper compartment and lower compartment (Dataset EV3 and EV4). *Nnat* is more strongly expressed in the lower third ventricle (Fig 4F—left), and its expression pattern coincides with the ependymal cell layer that lines the ventricular system. By contrast, the expression of *Enpp2* was located in the upper third ventricle (Fig 4F—right) and is absent from the ependymal cell layer. The third ventricle is highlighted in red. Interestingly, we also found a distinct cluster of ependymal cells lining the third ventricle (Fig 4D).

In Slide‐seq V2 embryo data, the developing eye displayed subtle transcriptional patterning (Fig 4G). Anterior Lens Epithelial Cells are characterized by two distinct spatial patterns organized in a concentric fashion. We observed a similar effect in Lens Vesicle cell distribution. We compared gene expression between Anterior Lens Epithelial cell layers and found 81 differentially expressed genes (Dataset EV2). For example, *Cryba4* was highly expressed in the inner layer while *Ccnd2* was expressed in the outer layer (Fig 4H—top row). Similarly, differential gene expression analysis between Lens Vesicle cells returned 74 differentially expressed genes including *Pmel* and *Aldh1a1* (Fig 4H).

While it remains unclear if these shifts represent novel cell types in the developing eye or spatially resolved developmental cues, Vesalius provides an easy, robust, and reproducible way of accessing this information via territory isolation in high‐resolution ST.

### Cells show territory‐specific gene expression in the mouse hippocampus

**Figure 5:**
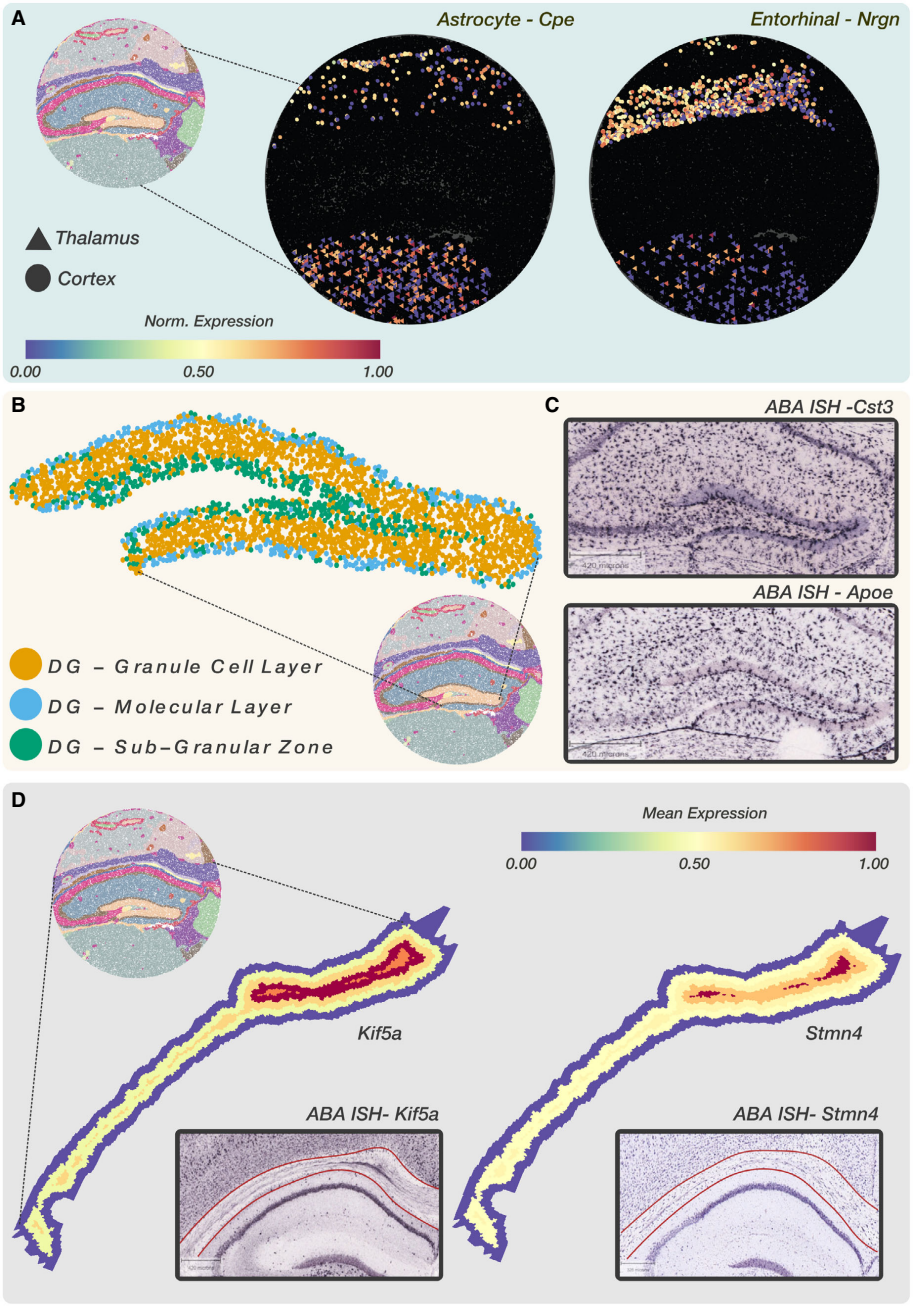
Investigation of territories reveals gene expression patterns linked to neighboring tissues and tissue morphology ADifferential gene expression analysis between cells contained in the cortex and the thalamus shows that spatial location influences gene expression. Astrocytes in the cortex are enriched with *Cpe*, while entorhinal cells in the thalamus are enriched with *Nrgn*.BBarcode clustering of the isolated dentate gyrus reveals transcriptional dissimilarity between each dentate gyrus (DG) layer.CDifferential gene expression analysis between DG Granule cell layer and DG sub‐granular zone displayed a high expression of *Cst3* and *Apoe* at the border between layers. *Cst3* and *Apoe* border expression is corroborated by Allen brain Atlas ISH images.DLayered expression pattern of *Stmn4* and *Kif5a* within the isolated corpus callosum showed a higher expression at the center of the corpus callosum. ISH images corroborate the spatial expression pattern of both genes. Corpus callosum contained within red lines.

Territory isolation enables us to study territory‐specific genes for the same cell type. To ensure that we are only accounting for the transcriptome of a single cell type, we first ran Robust Cell Type Decomposition (Cable *et al*, 2021) (RCTD) on Slide‐seq V2 mouse hippocampus data (see Materials and Methods). We selected all barcodes (*n* = 12,013) that contained a single cell type or homotypic spots and used these annotations to assign cell types across the mouse hippocampus. Next, we isolated territories corresponding to the cortex (1 to 6 and 8 in Fig 3A) and thalamus (28 and 33 in Fig 3A). We used Vesalius to compare cells that appear in both territories (> 30 cells) and discovered 73 differentially expressed genes (*P* < 0.01—Wilcoxon rank‐sum test) across four cell types (astrocytes, interneuron, oligodendrocytes, and entorhinal cells) (Dataset EV4). We illustrate the differential expression of *Cpe* in Astrocytes and *Nrgn* in entorhinal cells in Fig 5A. Astrocytes exhibit a stronger expression of *Cpe* in the thalamus compared with the cortex, while entorhinal cells show a higher expression of *Nrgn* in the cortex.

### Investigation of territories reveals gene expression patterns linked to neighboring tissues and tissue morphology

We also tested whether gene expression patterns could be linked to the morphology of a territory. Each territory provided by Vesalius can be manipulated using morphological operators (see Materials and Methods) or even divided into layers. The use of morphological operators for example permits the inclusion of additional barcodes that are part of the neighboring tissue. A territory divided into layers can be used to compare gene expression between the center of the structure and the edge of the structure.

Inflation and barcode clustering of the dentate gyrus (DG‐GCL) revealed that this territory included a thin layer of barcodes belonging to the Dentate Gyrus—sub‐granular zone (DG‐SGZ) and the dentate gyrus—molecular layer (DG‐ML) (Fig 5B). Differential gene expression analysis displayed two genes *Cst3* and *Apoe* expressed at the border between the DG‐GCL and the DG‐SGZ (see Dataset EV3). Allen Brain Atlas ISH images corroborate these results by displaying a higher expression of both genes at the border of the DG‐GCL and the DG‐SGZ (Fig 5C). While increased expression of *Cst3* and *Apoe* could result from a high cellular density at the border, our results demonstrate how Vesalius can aid in recovering subtle spatially driven gene expression or cellular patterns at the border between tissues.

We also isolated, dilated, and performed a layer analysis of the corpus callosum. Remarkably, we found high expression of *Stmn4* and *Kif5a* in the innermost layers (Fig 5D). Mean expression of both genes gradually increased toward the core of the corpus callosum. The ISH images for *Stmn4* taken from the Allen Brain Atlas showed a stripe of *Stmn4* expression (Fig 5D). *Kif5a* also shows a stripe and strong expression toward the center of the murine brain. The corpus callosum is highlighted in red. All differentially expressed genes are listed in the Appendix (Dataset EV3 and EV4).

## Discussion

Spatial transcriptomics—as a technique—has demonstrated its ability to recover the transcriptome of cells within the context of a tissue (Rao *et al*, 2021). This technical advancement has highlighted the influence of spatial context and cellular microenvironment on the transcriptome of cells (Hunter *et al*, 2021). Alongside experimental development, computational frameworks have been developed to analyze these new data sets (Satija *et al*, 2015; preprint: Pham *et al*, 2020; Dries *et al*, 2021; Hu *et al*, 2021; preprint: Peng *et al*, 2021; Zhao *et al*, 2021; preprint: Fu *et al*, 2021a; Dong & Zhang, 2022; Palla *et al*, 2022). However, it remains challenging to accurately recover spatial domains especially when these domains contain a multitude of cell types. Here, we present Vesalius—an R package to effectively perform *in silico* anatomization of high‐resolution ST data. Vesalius provides a reproducible framework for the isolation and in‐depth analysis of tissue territories.

The key to Vesalius's effectiveness in recovering heterogenous tissue territories resides in the embedding of the transcriptome into image arrays (Figs 1A and EV1A). The application of image processing techniques to these RGB images retrieves tissue territories without any assumption about the transcriptome of neighboring cells. In this context, an anatomical structure is characterized by its transcriptome and overall cell composition. Taken together, Vesalius can easily segment tissue territories containing heterogenous cell populations (Figs 2A–D and 3). In contrast, computational tools such as BayesSpace (Zhao *et al*, 2021) and Giotto (Dries *et al*, 2021) assume that neighboring spots are likely to be part of the same cluster if they are transcriptionally similar. While this assumption holds in lower resolution or in homogenous tissues, high‐resolution ST data sets recover the cellular complexity of tissues and thus neighboring spots are not always guaranteed to be of the same cell type.

We demonstrate the challenge of dealing with heterogenous territories by benchmarking BayesSpace (Zhao *et al*, 2021), STAGATE (Dong & Zhang, 2022), SpaGCN (Hu *et al*, 2021), SEDR (preprint: Fu *et al*, 2021a), Giotto (Dries *et al*, 2021), Seurat (Satija *et al*, 2015), and Vesalius in simulated data sets. Our simulations were designed to include different scenarios that could arise from real high‐resolution ST data (Fig EV1C). Overall, Vesalius is the highest performing algorithm across all regimes (Fig 2A–D). BayesSpace did not identify clear separations between territories once heterogeneity was introduced. In all cases, Giotto was unable to recover any clear territory even in the pure regime. Seurat also falters, but this reflects the fact that Seurat was never designed to recover spatial domains accurately but rather cluster similar transcriptomes together. STAGATE and SpaGCN performed reasonably well at recovering simulated tissue territories; however, both tools tend to overestimate the number of territories present. The performance of SEDR was low in most regimes, but the drop in performance was most notable in highly heterogenous simulation regimes. Vesalius can recover territories much more accurately, especially in cases where a territory contains multiple cell types, or each territory is characterized by a shift in cell‐type proportion. While Vesalius does tend to merge territories together in our dotted simulations, we demonstrate that isolation of uniform territories in real ST data provides a convenient way to investigate the finer details of ST data.

Vesalius shows a strong ability to recover tissue territories in high‐resolution ST data such as Slide‐seq V2 in both mouse hippocampus and mouse embryo (Fig 3A–C). Territories are uniform and provide tissue territories that may contain multiple cell types. BayesSpace can recover anatomical structures in Slide‐Seq V2 as long as these structures are composed of similar cell types (Figs 3B–D and EV2, EV3, EV4). These results are comparable to those produced with Seurat (Fig 3B–D and EV3, EV4, EV5) that does not consider the spatial component at all and simply maps cell cluster to their respective locations. The current trend in sequencing‐based ST is to increase resolution (Vickovic *et al*, 2019; Liu *et al*, 2020; Cho *et al*, 2021; preprint: Fu *et al*, 2021b), and achieving sub‐cellular resolution will prove extremely challenging to analyze whether heterogeneity is not considered carefully. We demonstrate the broad applicability of Vesalius in high‐resolution ST by recovering tissue territories in both Seq‐Scope (Cho *et al*, 2021) and seqFISH (Lohoff *et al*, 2021) data sets (Fig 3E–G). This application of Vesalius can also be successfully performed in lower‐resolution data sets such as Visium 10X (Ståhl *et al*, 2016; Fig 3H–J).

Isolation of spatial territories enhances the analysis of ST data. The clusters provided by BayesSpace and Seurat do not provide a convenient way of comparing spatial territories (Fig EV2, EV3, EV4, EV5), and sub‐clustering does not equate to spatial domain analysis due to the widespread of barcodes within clusters. We exemplify how the isolation of uniform territories provides an easy and reproducible way of investigating the finer details of spatial patterning. The CA2 field is often lost and merged with the CA1 and CA3 field (Fig 3B), yet we were able to recover all three CA fields in the mouse hippocampus after territory isolation (Fig 4A). The isolation of territories ensures that weaker expression patterns such as the expression of *Pcp4*—a CA2 field marker—are not lost in favor of stronger ones (Fig 4B and C). This approach also illustrated that the medial habenula and third ventricle were in fact divided into compartments (Fig 4D). For example, we showed that 119 genes are differentially expressed between medial habenula compartments with the expression of *Gabbr2* and *Calb2* delineating the position of both compartments in ISH images taken from the Allen Brain Atlas (Fig 4E). The isolation of the embryonic eye (Fig 4G and H) displayed transcriptional shifts that could either indicate novel cell types or spatially resolved developmental cues occurring during development. Finally, we were able to use isolated territories and demonstrate that cells in the mouse hippocampus exhibit territory‐specific gene expression patterns: Astrocytes in the cortex showed a higher expression of *Cpe* compared with astrocytes in the thalamus (Fig 5A). These results demonstrate how crucial spatial context is to understand the transcriptome of cells and how it can provide a new way of annotating cell types based on their spatial location.

Vesalius's unique way of representing territories also lends itself to the manipulation of territories and investigating gene expression in the context of neighboring tissues. For instance, we identified an increased expression of *Cst3* and *Apoe* at border of the DG‐GCL and DG‐SGZ (Fig 5B and C). We also discovered a heightened expression of *Kif5a* and *Stmn4* at the center of the corpus callosum (Fig 5D).

Finally, the concept of embedding the transcriptome into images offers the possibility to leverage powerful tools and concept developed in the field of computer vision. We hope that Vesalius will provide a new avenue for the investigation of ST data. Overall, Vesalius is an effective tool to recover spatial domains in high‐resolution ST data and isolate territories for further analysis. We demonstrate that the isolation of spatial domains uncovers the finer details of spatial patterning and capitalizes on the wealth of information contained in high‐resolution ST data. Vesalius complements other ST analysis methods, enhances potential biological insights, and may help in providing an additional layer to cell‐type annotation based on their spatial location.

## Materials and Methods

### Reagents and Tools table

**Table:** 

Software	Reference or source
R programming Language (4.0.3)	https://www.r‐project.org/
Anaconda3‐2022.05‐Linux‐x86_64	https://www.anaconda.com/products/distribution
Vesalius 1.0.1	https://patrickcnmartin.github.io/Vesalius/
Seurat 4.0.1	https://satijalab.org/seurat/
STAGATE 1.0.1	https://github.com/QIFEIDKN/STAGATE
SEDR	https://github.com/JinmiaoChenLab/SEDR
SpaGCN 1.2.5	https://github.com/jianhuupenn/SpaGCN
BayesSpace (Modified from 1.3.1)	https://github.com/patrickCNMartin/BayesSpace
Giotto 1.1.0	https://github.com/RubD/Giotto
RCTD (Spacexr)	https://github.com/dmcable/spacexr
mclust 5.4.7	https://cran.r‐project.org/web/packages/mclust/index.html
mcclust 1.0	https://cran.r‐project.org/web/packages/mcclust/index.html
ggpubr_0.4.0	https://cran.r‐project.org/web/packages/ggpubr/index.html

### Methods and Protocols

#### Preprocessing

Preprocessing of data prior to Vesalius image building was handled by the Seurat package (Satija *et al*, 2015). Data sets were loaded, log normalized, and scaled using the default Seurat settings. Variable features (*n* = 2,000) were extracted prior to PCA. Log normalization was preferred for preprocessing as it would provide a fair comparison with other tools and methods. Selecting the number of principle components used for analysis can be achieved by using the Seurat *JackStraw* function or the Seurat *ElbowPlot* function.

#### Cell‐type deconvolution

To infer the cell‐type identity of individual beads in Slide‐seq V2 data sets, RCTD (Robust Cell Type Decomposition) was used (Cable *et al*, 2021). RCTD is an R package that decomposes the cell‐type mixtures of spatial beads at single‐cell resolution. RCTD is trained with pre‐labeled single‐cell RNA‐sequencing (scRNA‐seq) data sets predicts the cell types of each bead. We selected RCTD as a deconvolution tool as it was purposefully designed for fine‐resolution spatial transcriptomics data such as Slide‐seq. We conducted simulation under doublet mode, which is recommended for Slide‐seq data sets (Cable *et al*, 2021). It assumes that each bead contains up to two different cell types. We trained RCTD using scRNA‐seq data set for hippocampus (Saunders *et al*, 2018) that consists of 27,953 genes and 113,507 cells annotated with 17 cell types. The scRNA‐seq data set was obtained from the publicly available repository, DropViz. The results of the cell‐type decomposition were validated by cell‐type markers accompanied with the scRNA‐seq data set from hippocampus (preprint: Kim *et al*, 2022).

#### Vesalius—Transcriptome embedding into the RGB color space

To embed the transcriptome into the RGB color space, Vesalius first computes principal component analysis (PCA) followed by a 3‐dimensional Uniform Manifold Approximation and Projection (UMAP). UMAP latent space is min‐max normalized for each barcode B_i_:

(1) $${B^{\prime }}_{i}=\frac{B_{i}-\min\left(B\right)}{\max\left(B\right)-\min\left(B\right)}.$$

The normalized 3‐dimensional space can simply be converted into an RGB color space (Appendix Fig S1A).

We used the default 30 PCs for UMAP projections. Alternatively, Vesalius can directly use PCA loading values in which case Vesalius selects a PC slice composed of three principal components—one for each color channel (RGB). For each color channel c and for each barcode B_i_, Vesalius takes the sum of the absolute value of all loading values associated with B_i_ in color channel c:

(2) $${B^{\prime }}_{i}^{c}=\sum_{k=1}^{n}|L_{k}|$$

With *k* = 1…*n* and *n* being the number of non‐zero loading values *L* associated with barcode ${B^{\prime }}_{i}^{c}$.

For each color channel, Vesalius returns a numerical array of length equal to the number of barcodes. To ensure that these values are within color space bounds, each color channel is min‐max normalized. As three PCs are selected in a “slice” (e.g., PC1, PC2, and PC3) and RGB color space contains three dimensions, Vesalius simply assigns the color value obtained from each PC into each color space dimension (red, green, and blue—Fig EV1A).

### Vesalius—Building images arrays from spatial transcriptomics

Each ST data set contains a set of coordinates describing the x and y position of each barcode/spot. As the coordinates will not necessarily be uniformly distributed and contiguous, Vesalius expands punctual coordinate values into tiles using Voronoi tessellation and fills each tile using rasterization. First, we filter stray barcodes by comparing barcode density in a grid covering the entire ST assay and removing any barcodes that fall into a grid section with a low barcode density (determined based on quantiles of grid density). Next, we produce a Voronoi diagram using the remaining barcodes. Each tile is then converted into a pixel set via rasterization. The color code obtained for each barcode is then assigned to its tile. RGB image arrays are three‐dimensional arrays, and as such each color channel in the image will receive a distinct set of color value for each latent space dimension (UMAP or PCA). The tiles associated with each barcode remain the same between color channels. Optionally, Vesalius can resize the image output using nearest‐neighbor interpolation as default. All barcodes will be retained after image resizing.

#### Vesalius—Image processing and segmentation

Image arrays are handled internally by the *imager* (Barthelmé & Tschumperlé, 2019) and *imagerExtra* R packages, which contain a set of image processing methods such as blurs, segmentation, and image manipulation. Images can be regularized with nonlinear total variation‐based noise removal algorithms (Rudin *et al*, 1992). Vesalius provides an iterative segmentation approach to reduce the color space of an image and extract territories.

First, the image array is smoothed using either Gaussian blur, median blur, box blur, or a combination of the aforementioned methods. Second, color values are clustered in *k* clusters using K‐means clustering. The choice of *k* is left to the user and will depend on the level of territory refinement the user wishes to have. Multiple rounds of smoothing and segmentation may be applied. In the instance that multiple values of *k* are used, Vesalius will smooth and segment the image as described above and repeat the process for all values of *k*. A decreasing *k* value at each round will iteratively decrease the color space.

To decrease computational time, the clustering is only applied to the center pixel value. The center pixel is defined by the pixel corresponding to the original barcode location before tessellation and rasterization. Vesalius also provides the option to smooth and segment images using all pixels instead. Using all pixels for segmentation produces sharper segments between homogenous territories. However, this sharpness in homogenous territories may come at the cost of increased noise in heterogenous territories.

#### Vesalius—Isolating territories

Once barcodes have been assigned to a color cluster, each color cluster is further subdivided into territories. For every barcode present in a color cluster, Vesalius checks for all barcodes that are within a certain capture radius of each other and assigns them to a territory (Fig EV1B). This process is repeated for all barcodes in a color cluster until they have all been assigned to a territory. Vesalius repeats this process for each color cluster. The capture radius is defined as the distance value that corresponds to the proportion of maximum distances between all barcodes in the ST assay. First, Vesalius computes a distance matrix between all barcodes in 2D space and extracts the maximum possible distance between barcodes. A user‐selected parameter defines the proportion of this maximum distance that should be used as a capture radius. Territories may be assigned to the isolated group. This group is defined by territories that did not contain enough cells (user‐defined parameter) and outside of the capture radius (user defined).

While the number of color clusters is fixed by *k* (or final *k*) as described above, the final number of territories may vary depending on parameter selection used throughout the analysis. This includes image processing and territory isolation. The selection of these parameters depends on the users’ interests and how they wish to explore ST data. In the context, the value of *k* serves a similar role as the granularity resolution in Louvain or Leiden clustering.

#### Simulating high‐resolution ST data and tissue heterogeneity

To benchmark Vesalius in high‐resolution ST data sets containing heterogenous tissues, we simulated ST data using Slide‐seq V2 after cell‐type deconvolution. First, we applied RCTD (Cable *et al*, 2021) to deconvolute cell types and selected barcodes that contained a single cell type (*n* = 12,013). From these barcodes, we selected cell types that contained at least 50 different barcodes leaving 11,957 barcodes across 13 cell types. This approach ensures that we more closely mimic the biology of heterogenous tissue rather than ST assay technical limitations. Next, we generated 6,000 random x and y coordinate pairs that will be assigned to a territory. For each territory, we randomly sampled cell types and barcodes under four different regimes: pure, uniform, exponential, and dotted (Fig EV1C). The pure regime contains a single randomly sampled cell type in each territory and randomly selected barcodes for that cell type. The uniform regime contains *n* cell types in equal proportion. Each territory contains different sets of randomly sampled cell types. The exponential regime contains n cell types in unequal proportion:

(3) $$P_{l}=\frac{e^{l}}{\sum_{l=1}^{n}e^{l}}$$

where *n* is the number of different cell types and P_l_ is the proportion of cell type *l*. Each territory contains the same cell types in different proportions. The pure, uniform, and exponential regimes are divided into three equally sized territories. Finally, the dotted regime contains a background territory in which five circular territories are placed. The radius of each territory is randomly selected, and the center of each territory is placed upon a randomly selected coordinate pair. Each territory including the background contains a random number of cell types (between 1 and 3 cell types), and for each cell type, we randomly sample barcodes. To further increase the complexity of the dotted regime, we also allow for overlaps between territories. While the input parameters to our simulation should produce six total territories, allowing overlaps means that some territories may completely mask another. To reduce any potential cell‐type bias, we ran the simulation 10 times each time randomly selecting different cell types and different barcodes. The simulated data set produced at each sampling round was the same between each tool, and a summary of the cell types sampled at each round can be found in Dataset EV1. The code used to run simulations and method comparison (see below) can be found here: https://github.com/patrickCNMartin/Vesalius/tree/main/methodComp.

#### Method comparison

We compared the performance of Vesalius to the performance of other methods such as Seurat (Satija *et al*, 2015), BayesSpace (Zhao *et al*, 2021), SpaGCN (Hu *et al*, 2021), SEDR (preprint: Fu *et al*, 2021a), STAGATE (Dong & Zhang, 2022), and Giotto(Dries *et al*, 2021) in our simulated data sets.

BayesSpace required a minor modification to accommodate Slide‐seq V2 as well as our simulated data sets. We adapted the .*find_neighbors* function to select neighbors based on Euclidean distance in 2D space and select the 6 closest neighbors. We added a new platform named “SS” for Slide‐Seq. The forked and modified version of BayesSpace is available at: https://github.com/patrickCNMartin/BayesSpace.

Simulated data provide ground‐truth data sets that can be used to compare each tool in high‐resolution data sets. We assessed each tool's ability to recover each territory using and Adjusted Rand Index (Rand, 1971) and variation of information metric (Meilǎ, 2007). ARI scores are affected by the granularity of the clustering and the variation of information metric provides an alternative measure of performance. It is important to acknowledge that even though Seurat is consistently used for spatial domain retrieval benchmarking, Seurat is not designed to retrieve spatial domains and is expected to perform poorly in highly heterogenous tissues.

Statistical analysis was performed to compare overall performance between methods over 10 simulation replicates. We first tested for normality using a Shapiro–Wilks test, and then we tested the homogeneity of variance with a Bartlett test using base R functionality. We compared all groups with a Kruskal–Wallis test and used a Wilcoxon rank‐sum test for multiple comparisons. *P*‐values for the Kruskal–Wallis are shown in the figure (Fig 2A–C), and multiple comparisons are shown using start notation for clarity. Comparisons were carried out using the *ggpubr* R package.

All code related to the comparison between methods can be found here: https://github.com/patrickCNMartin/Vesalius/tree/main/methodComp

#### Vesalius—Differential gene expression and territory markers

Marker genes and differentially expressed genes can be extracted from each territory. This process can be carried out in a fivefold manner:

- Territory vs all other territories combined
- Territory vs all other territories individually
- Territory(ies) vs territory(ies)
- Cells within a territory(ies) vs cells within a territory(ies)
- Layer in territory vs layer in territory

To be considered for differential expression analysis, genes must pass a set of criteria. First, genes must be present in a certain percentage of barcodes in at least one territory (> 10% of beads as default). Second, the log fold change must be above a certain threshold (logFC ≥ 0.25). It should be noted that this threshold is applied in the case of upregulation as well as downregulation. Remaining genes are tested for significant differential gene expression by using a Wilcoxon rank‐sum test (Bonferroni corrected P‐value < 0.05). Gene expression patterns can be visualized by using the *viewGeneExpression* function provided in the Vesalius package. Gene expression can be visualized over the entire slide or in an isolated territory. For visualization, gene expression is min‐max normalized.

#### Vesalius—Territory dilation, erosion, filling, and cleaning

By using image representation of territories, Vesalius provides a convenient way to manipulate territories using image morphology. Vesalius encompasses dilation, erosion, filling, and cleaning into a single function. We summarize image morphologies using a “morphology factor” described by an array of integers. Positive integers increase territory size, while negative integers decrease territory size. Numerical arrays of positive and negative integers provide filling and cleaning morphologies. For example, in the case of a cleaning morphological operator, a morphology factor array v = [−5,5] will first erode the territory by 5 pixels and then dilate the territory by 5 pixels.

#### Vesalius—Territory layering and layered gene expression

Isolated territory layering is achieved by capturing territory edges and removing barcodes belonging to the edge and repeating the process until no more barcodes remain. First, the isolated territory is converted to a black and white image and X‐Y Sobel edge detection is applied. All barcodes that share a pixel with the detected edge are pooled into a layer and removed from the territory. The edge detection and pooling process are repeated until all barcodes have been assigned to a layer. Layers can be combined by merging neighboring layers together (Fig EV1D). Differential gene expression between layers is carried out using a Wilcoxon rank‐sum test (Bonferroni corrected P‐value < 0.05 & logFC ≥ 0.25). Visualization of gene expression between layers is provided by the viewLayeredExpression in the Vesalius package. Layers are described by their normalized and averaged expression values.

#### Cell clustering and annotation

Clustering analysis of isolated territories was carried out using the Seurat package. Clusters and territories were manually annotated using their respective genetic markers. Markers were extracted from each cluster using the *FindAllMarkers* function provided by Seurat and the *extractClusterMarkers* function provided by Vesalius. *FindAllMarkers* compares clusters between each other (in the isolated territory), while *extractClusterMarkers* compares clusters to all other barcodes present in the slide. This distinction ensures that we recover subtle differences between cell types and territory‐specific gene expression.

We used the default Wilcoxon rank‐sum test for marker extraction. Identified markers were compared with Allen Brain Atlas (Lein *et al*, 2007) (https://mouse.brain‐map.org/), the lifeMaps/geneCard database (Edgar *et al*, 2013) (https://discovery.lifemapsc.com/in‐vivo‐development), or panglaodb database (Franzén *et al*, 2019) (https://panglaodb.se/) to assign cell type to clusters and territories. Manual annotation of cell clusters was preferred over automated methods to ensure correct tissue annotations, rare cell‐type annotation and finally to maintain subtle spatially driven patterning. Cell‐type markers used for annotation are available in Dataset EV2.

#### Analyzed publicly available datasets

Slide‐seq V2: Single Cell Portal. https://singlecell.broadinstitute.org/single_cell/study/SCP815/highly‐sensitive‐spatial‐transcriptomics‐at‐near‐cellular‐resolution‐with‐slide‐seqv2#study‐summary. Seq‐Scope: Deepblue. https://deepblue.lib.umich.edu/data/concern/data_sets/9c67wn05f. seqFISH: https://content.cruk.cam.ac.uk/jmlab/SpatialMouseAtlas2020/. Visium 10X: https://www.10xgenomics.com/resources/datasets. ISH Images: Allen Brain Atlas. https://mouse.brain‐map.org/. Reference Single cell: DropViz. http://dropviz.org/

## Author contributions

**Patrick C N Martin:** Conceptualization; software; formal analysis; investigation; visualization; methodology; writing – original draft; writing – review and editing. **Hyobin Kim:** Formal analysis; investigation; methodology; writing – review and editing. **Cecilia Lövkvist:** Formal analysis; investigation; writing – review and editing. **Byung‐Woo Hong:** Methodology; writing – review and editing. **Kyoung Jae Won:** Conceptualization; supervision; funding acquisition; methodology; writing – original draft; project administration; writing – review and editing.

In addition to the CRediT author contributions listed above, the contributions in detail are:

The project was conceptualized by PCNM and KJW. The methodology was developed by PCNM, BWH, and KJW. Investigation and formal analysis were carried out by PCNM, HK, and CL. Visualization was carried out by PCNM. Funding was acquired by KJW. Projection administration and supervision were handled by KJW. The writing of the original draft was carried out by PCNM and KJW. Reviews and editing were done by PCNM, HK, CL, BWH, and KJW.

## Disclosure and competing interests statement

The authors declare that they have no conflict of interest.

## Supporting information




Appendix

EVFigs

Dataset EV1

Dataset EV2

Dataset EV3

Dataset EV4

## Data Availability

The modified version of BayesSpace to accommodate Slide‐seqV2: GitHub. https://github.com/patrickCNMartin/BayesSpace. Vesalius: GitHub. https://patrickcnmartin.github.io/Vesalius/index.html. Vesalius Analysis: GitHub. https://patrickcnmartin.github.io/Vesalius/articles/Vesalius_Analysis/Vesalius_analysis.html. Vesalius Method Comparison and Simulation: GitHub. https://github.com/patrickCNMartin/Vesalius/tree/main/methodComp.

## References

[msb202211080-bib-0001] Barthelmé S , Tschumperlé D (2019) Imager: an R package for image processing based on CImg. J Open Source Softw 4: 1012

[msb202211080-bib-0002] Bergenstråhle J , Larsson L , Lundeberg J (2020) Seamless integration of image and molecular analysis for spatial transcriptomics workflows. BMC Genomics 21: 1–7 10.1186/s12864-020-06832-3 PMC7386244 32664861

[msb202211080-bib-0003] Burgess DJ (2019) Spatial transcriptomics coming of age. Nat Rev Genet 20: 317 30980030 10.1038/s41576-019-0129-z

[msb202211080-bib-0004] Cable DM , Murray E , Zou LS , Goeva A , Macosko EZ , Chen F , Irizarry RA (2021) Robust decomposition of cell type mixtures in spatial transcriptomics. Nat Biotechnol 40: 517–526 33603203 10.1038/s41587-021-00830-w PMC8606190

[msb202211080-bib-0005] Chen KH , Boettiger AN , Moffitt JR , Wang S , Zhuang X (2015) Spatially resolved, highly multiplexed RNA profiling in single cells. Science 348: aaa6090 25858977 10.1126/science.aaa6090 PMC4662681

[msb202211080-bib-0006] Cho C‐SS , Xi J , Si Y , Park S‐RR , Hsu J‐EE , Kim M , Jun G , Kang HM , Lee JH (2021) Microscopic examination of spatial transcriptome using seq‐scope. Cell 184: 3559–3572.e22 34115981 10.1016/j.cell.2021.05.010 PMC8238917

[msb202211080-bib-0007] Dong K , Zhang S (2022) Deciphering spatial domains from spatially resolved transcriptomics with an adaptive graph attention auto‐encoder. Nat Commun 13: 1739 35365632 10.1038/s41467-022-29439-6 PMC8976049

[msb202211080-bib-0008] Dries R , Zhu Q , Dong R , Eng CHL , Li H , Liu K , Fu Y , Zhao T , Sarkar A , Bao F *et al* (2021) Giotto: a toolbox for integrative analysis and visualization of spatial expression data. Genome Biol 22: 1–31 33685491 10.1186/s13059-021-02286-2 PMC7938609

[msb202211080-bib-0009] Edgar R , Mazor Y , Rinon A , Blumenthal J , Golan Y , Buzhor E , Livnat I , Ben‐Ari S , Lieder I , Shitrit A *et al* (2013) LifeMap discovery™: the embryonic development, stem cells, and regenerative medicine research portal. PLoS ONE 8: e66629 23874394 10.1371/journal.pone.0066629 PMC3714290

[msb202211080-bib-0010] Eng CHL , Lawson M , Zhu Q , Dries R , Koulena N , Takei Y , Yun J , Cronin C , Karp C , Yuan GC *et al* (2019) Transcriptome‐scale super‐resolved imaging in tissues by RNA seqFISH+. Nature 568: 235–239 30911168 10.1038/s41586-019-1049-y PMC6544023

[msb202211080-bib-0011] Franzén O , Gan LM , Björkegren JLM (2019) PanglaoDB: a web server for exploration of mouse and human single‐cell RNA sequencing data. Database (Oxford) 2019: baz046 30951143 10.1093/database/baz046 PMC6450036

[msb202211080-bib-0012] Fu H , Xu H , Chong K , Li M , Ang KS , Lee HK , Ling J , Chen A , Shao L , Liu L *et al* (2021a) Unsupervised spatially embedded deep representation of spatial transcriptomics. *bioRxiv* 10.1101/2021.06.15.448542 [PREPRINT] PMC10790257 38217035

[msb202211080-bib-0013] Fu X , Sun L , Chen JY , Dong R , Lin Y , Palmiter RD , Lin S , Gu L (2021b) Continuous polony gels for tissue mapping with high resolution and RNA capture efficiency. *bioRxiv* 10.1101/2021.03.17.435795 [PREPRINT]

[msb202211080-bib-0014] Gerber KJ , Dammer EB , Duong DM , Deng Q , Dudek SM , Seyfried NT , Hepler JR (2019) Specific proteomes of hippocampal regions CA2 and CA1 reveal proteins linked to the unique physiology of area CA2. J Proteome Res 18: 2571–2584 31059263 10.1021/acs.jproteome.9b00103 PMC7039248

[msb202211080-bib-0015] Gurcan MN , Boucheron LE , Can A , Madabhushi A , Rajpoot NM , Yener B (2009) Histopathological image analysis: a review. IEEE Rev Biomed Eng 2: 147–171 20671804 10.1109/RBME.2009.2034865 PMC2910932

[msb202211080-bib-0016] Hu J , Li X , Coleman K , Schroeder A , Ma N , Irwin DJ , Lee EB , Shinohara RT , Li M (2021) SpaGCN: integrating gene expression, spatial location and histology to identify spatial domains and spatially variable genes by graph convolutional network. Nat Methods 18: 1342–1351 34711970 10.1038/s41592-021-01255-8

[msb202211080-bib-0017] Hunter MV , Moncada R , Weiss JM , Yanai I , White RM (2021) Spatially resolved transcriptomics reveals the architecture of the tumor‐microenvironment interface. Nat Commun 12: 1–16 34725363 10.1038/s41467-021-26614-z PMC8560802

[msb202211080-bib-0018] Kim H , Lövkvist C , Martin P , Kim J , Won KJ (2022) Detecting cell contact‐dependent gene expression from spatial transcriptomics data. *bioRxiv* 10.1101/2022.02.16.480673 [PREPRINT] PMC10632736 37815040

[msb202211080-bib-0019] Kurc T , Bakas S , Ren X , Bagari A , Momeni A , Huang Y , Zhang L , Kumar A , Thibault M , Qi Q *et al* (2020) Segmentation and classification in digital pathology for glioma research: challenges and deep learning approaches. Front Neurosci 14: 27 32153349 10.3389/fnins.2020.00027 PMC7046596

[msb202211080-bib-0020] Lein ES , Hawrylycz MJ , Ao N , Ayres M , Bensinger A , Bernard A , Boe AF , Boguski MS , Brockway KS , Byrnes EJ *et al* (2007) Genome‐wide atlas of gene expression in the adult mouse brain. Nature 445: 168–176 17151600 10.1038/nature05453

[msb202211080-bib-0021] Liu Y , Yang M , Deng Y , Su G , Enninful A , Guo CC , Tebaldi T , Zhang D , Kim D , Bai Z *et al* (2020) High‐spatial‐resolution multi‐omics sequencing via deterministic barcoding in tissue. Cell 183: 1665–1681.e18 33188776 10.1016/j.cell.2020.10.026 PMC7736559

[msb202211080-bib-0022] Lohoff T , Ghazanfar S , Missarova A , Koulena N , Pierson N , Griffiths JA , Bardot ES , Eng CHL , Tyser RCV , Argelaguet R *et al* (2021) Integration of spatial and single‐cell transcriptomic data elucidates mouse organogenesis. Nat Biotechnol 40: 74–85 34489600 10.1038/s41587-021-01006-2 PMC8763645

[msb202211080-bib-0023] Lubeck E , Coskun AF , Zhiyentayev T , Ahmad M , Cai L (2014) Single‐cell in situ RNA profiling by sequential hybridization. Nat Methods 11: 360–361 24681720 10.1038/nmeth.2892 PMC4085791

[msb202211080-bib-0024] Meilǎ M (2007) Comparing clusterings—an information based distance. J Multivar Anal 98: 873–895

[msb202211080-bib-0025] Palla G , Spitzer H , Klein M , Fischer D , Schaar AC , Kuemmerle LB , Rybakov S , Ibarra IL , Holmberg O , Virshup I *et al* (2022) Squidpy: a scalable framework for spatial omics analysis. Nat Methods 19: 171–178 35102346 10.1038/s41592-021-01358-2 PMC8828470

[msb202211080-bib-0026] Peng T , Chen GM , Tan K (2021) GLUER: integrative analysis of single‐cell omics and imaging data by deep neural network. *bioRxiv* 10.1101/2021.01.25.427845 [PREPRINT]

[msb202211080-bib-0027] Pham D , Tan X , Xu J , Grice LF , Lam PY , Raghubar A , Vukovic J , Ruitenberg MJ , Nguyen Q (2020) stLearn: integrating spatial location, tissue morphology and gene expression to find cell types, cell‐cell interactions and spatial trajectories within undissociated tissues. *bioRxiv* 10.1101/2020.05.31.125658 [PREPRINT]

[msb202211080-bib-0028] Rand WM (1971) Objective criteria for the evaluation of clustering methods. J Am Stat Assoc 66: 846–850

[msb202211080-bib-0029] Rao A , Barkley D , França GS , Yanai I (2021) Exploring tissue architecture using spatial transcriptomics. Nature 596: 211–220 34381231 10.1038/s41586-021-03634-9 PMC8475179

[msb202211080-bib-0030] Rodriques SG , Stickels RR , Goeva A , Martin CA , Murray E , Vanderburg CR , Welch J , Chen LM , Chen F , Macosko EZ (2019) Slide‐seq: a scalable technology for measuring genome‐wide expression at high spatial resolution. Science 363: 1463–1467 30923225 10.1126/science.aaw1219 PMC6927209

[msb202211080-bib-0031] Rudin LI , Osher S , Fatemi E (1992) Nonlinear total variation based noise removal algorithms. Physica D 60: 259–268

[msb202211080-bib-0032] Satija R , Farrell JA , Gennert D , Schier AF , Regev A (2015) Spatial reconstruction of single‐cell gene expression data. Nat Biotechnol 33: 495–502 25867923 10.1038/nbt.3192 PMC4430369

[msb202211080-bib-0033] Saunders A , Macosko EZ , Wysoker A , Goldman M , Krienen FM , de Rivera H , Bien E , Baum M , Bortolin L , Wang S *et al* (2018) Molecular diversity and specializations among the cells of the adult mouse brain. Cell 174: 1015–1030.e16 30096299 10.1016/j.cell.2018.07.028 PMC6447408

[msb202211080-bib-0034] Ståhl PL , Salmén F , Vickovic S , Lundmark A , Navarro JF , Magnusson J , Giacomello S , Asp M , Westholm JO , Huss M *et al* (2016) Visualization and analysis of gene expression in tissue sections by spatial transcriptomics. Science 353: 78–82 27365449 10.1126/science.aaf2403

[msb202211080-bib-0035] Stickels RR , Murray E , Kumar P , Li J , Marshall JL , Di Bella DJ , Arlotta P , Macosko EZ , Chen F (2021) Highly sensitive spatial transcriptomics at near‐cellular resolution with slide‐seqV2. Nat Biotechnol 39: 313–319 33288904 10.1038/s41587-020-0739-1 PMC8606189

[msb202211080-bib-0036] Vickovic S , Eraslan G , Salmén F , Klughammer J , Stenbeck L , Schapiro D , Äijö T , Bonneau R , Bergenstråhle L , Navarro JF *et al* (2019) High‐definition spatial transcriptomics for in situ tissue profiling. Nat Methods 16: 987–990 31501547 10.1038/s41592-019-0548-y PMC6765407

[msb202211080-bib-0037] Vu QD , Graham S , Kurc T , To MNN , Shaban M , Qaiser T , Koohbanani NA , Khurram SA , Kalpathy‐Cramer J , Zhao T *et al* (2019) Methods for segmentation and classification of digital microscopy tissue images. Front Bioeng Biotechnol 7: 53 31001524 10.3389/fbioe.2019.00053 PMC6454006

[msb202211080-bib-0038] Zhao E , Stone MR , Ren X , Guenthoer J , Smythe KS , Pulliam T , Williams SR , Uytingco CR , Taylor SEB , Nghiem P *et al* (2021) Spatial transcriptomics at subspot resolution with BayesSpace. Nat Biotechnol 39: 1–10 34083791 10.1038/s41587-021-00935-2 PMC8763026

[msb202211080-bib-0039] Zhuang X (2021) Spatially resolved single‐cell genomics and transcriptomics by imaging. Nat Methods 18: 18–22 33408406 10.1038/s41592-020-01037-8 PMC9805800

