# Supplementary material for: Vesalius: high‐resolution in silico anatomization of spatial transcriptomic data using image analysis
Source: Mol Syst Biol. 2022 Sep 6;18(9):e11080. doi: 10.15252/msb.202211080 (PMC9446088; doi:10.15252/msb.202211080)
Supplement: Supplementary file 1 — Appendix [file MSB-18-e11080-s003.pdf]

Vesalius: high-resolution in silico anatomization of Spatial Transcriptomic data using Image Analysis.

Appendix Figure

Table of Content

1. Appendix Figure S1 - UMAP projection and PCA loading value define similar yet not identical territories.
2. Appendix Figure S2 - Demonstrating the effects of image processing on Vesalius images.
3. Appendix Figure S3 - Validation of CA2 field Marker genes.

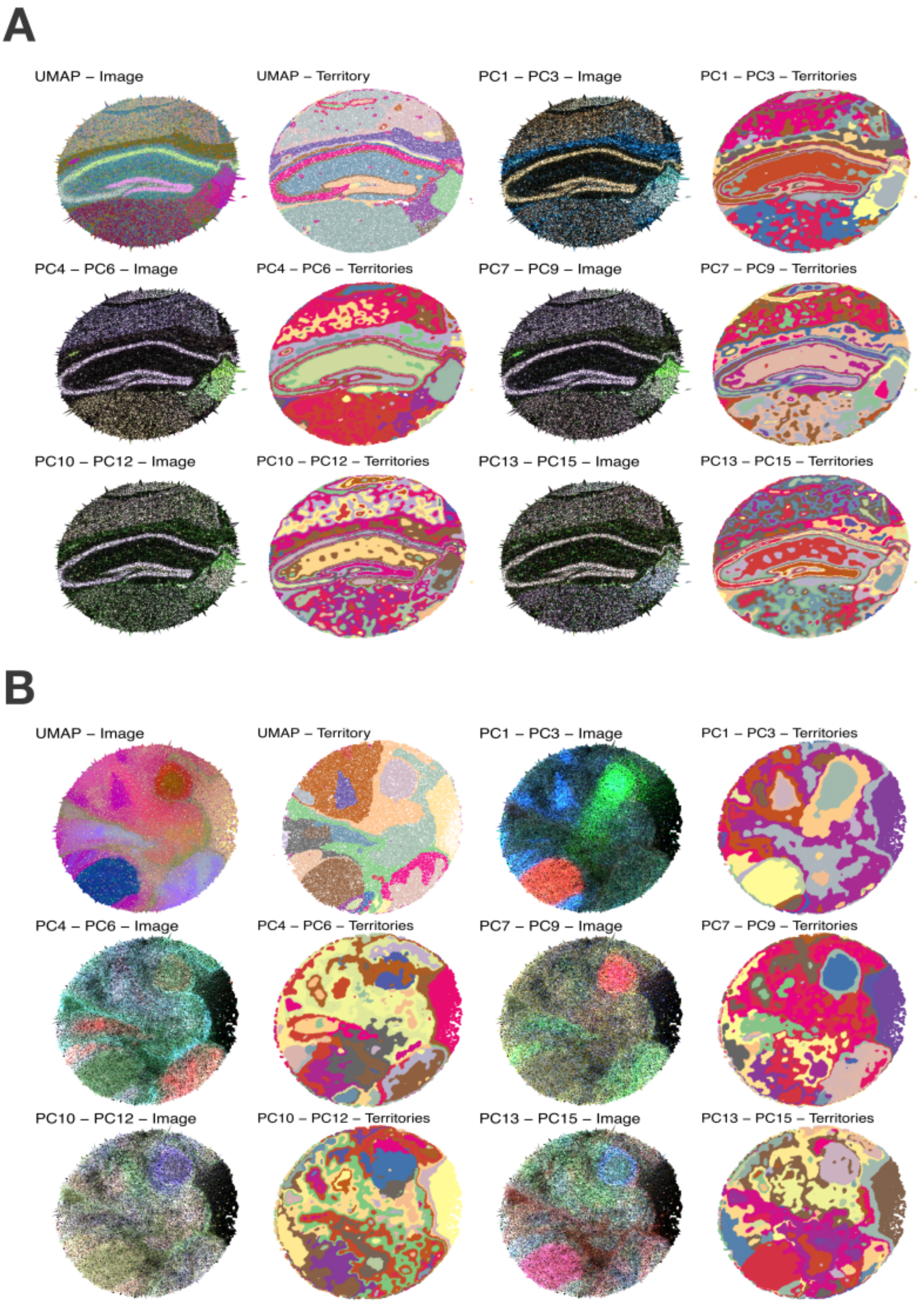

Appendix Figure S1 - UMAP projection and PCA loading value define similar yet not identical territories.

A UMAP and PCA slices in the mouse hippocampus (Puck_200115_08). Overall UMAP projections better recover the structure of the mouse hippocampus.

B UMAP and PCA slices in the mouse embryo (Puck_190926_03). While UMAP projections recover more uniform territories, PCA slices enable a more targeted selection of territories. The selection of territories depends on the interest of the user and PCA enables more flexibility in the choice of territories.

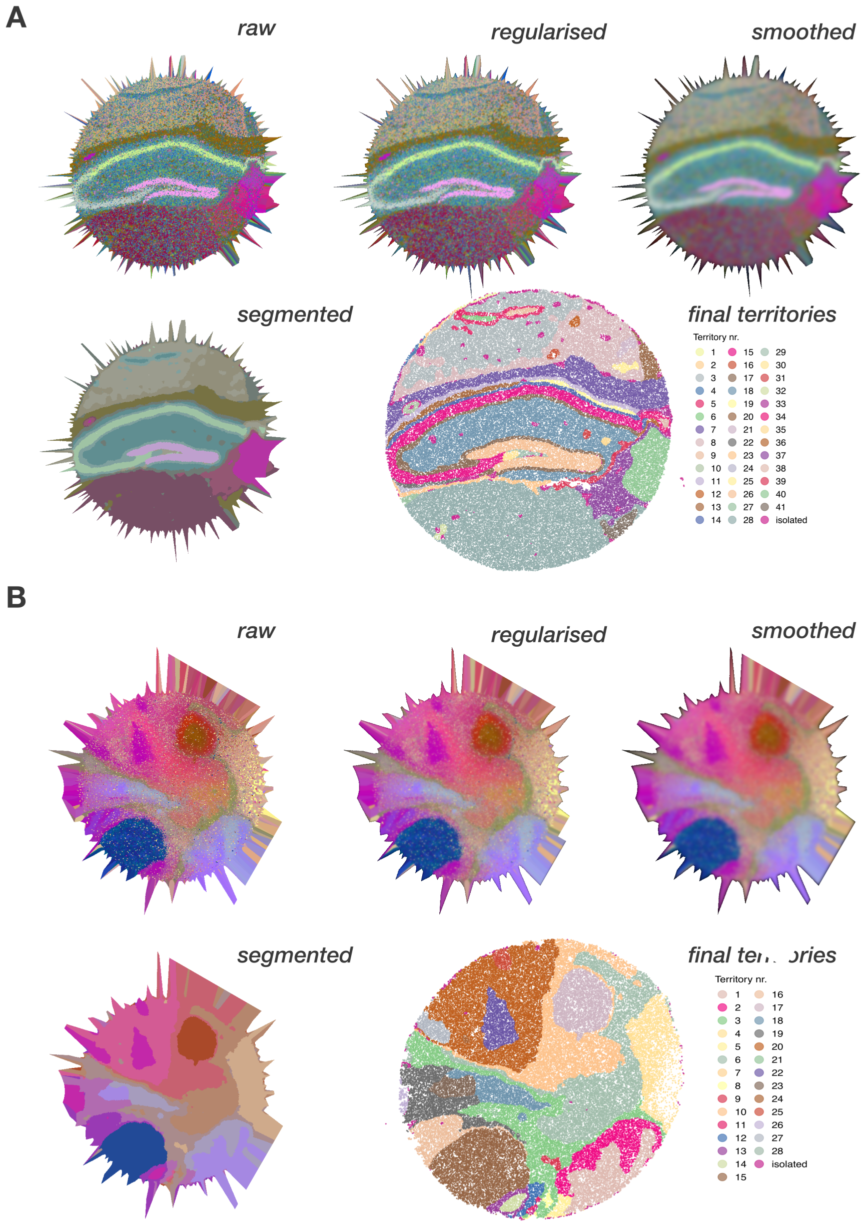

Appendix Figure S2 - Demonstrating the effects of image processing on Vesalius images.

A View of Vesalius images produced with Slide-seq V2 mouse hippocampus (Puck_200115_08). The raw image represents the color embeddings after running Vesalius with UMAP mode. Next, regularized shows the same image after regularization. The smoothed image shows the image after applying smoothing kernels (iso and box). The segmented image shows the results after k-means image segmentation. Finally, the resulting territories after isolating each element in the segmented image. Territories are represented in “false color” while raw, regularized and segmented are “true color” output.

B View of Vesalius images produced with Slide-seq V2 mouse embryo (Puck_190926_03). The raw image represents the color embeddings after running Vesalius with UMAP mode. Next, regularized shows the same image after regularization. The smoothed image shows the image after applying smoothing kernels (iso and box). The segmented image shows the results after k-means image segmentation. Finally, the resulting territories after isolating each element in the segmented image. Territories are represented in “false color” while raw, regularized and segmented are “true color” output.

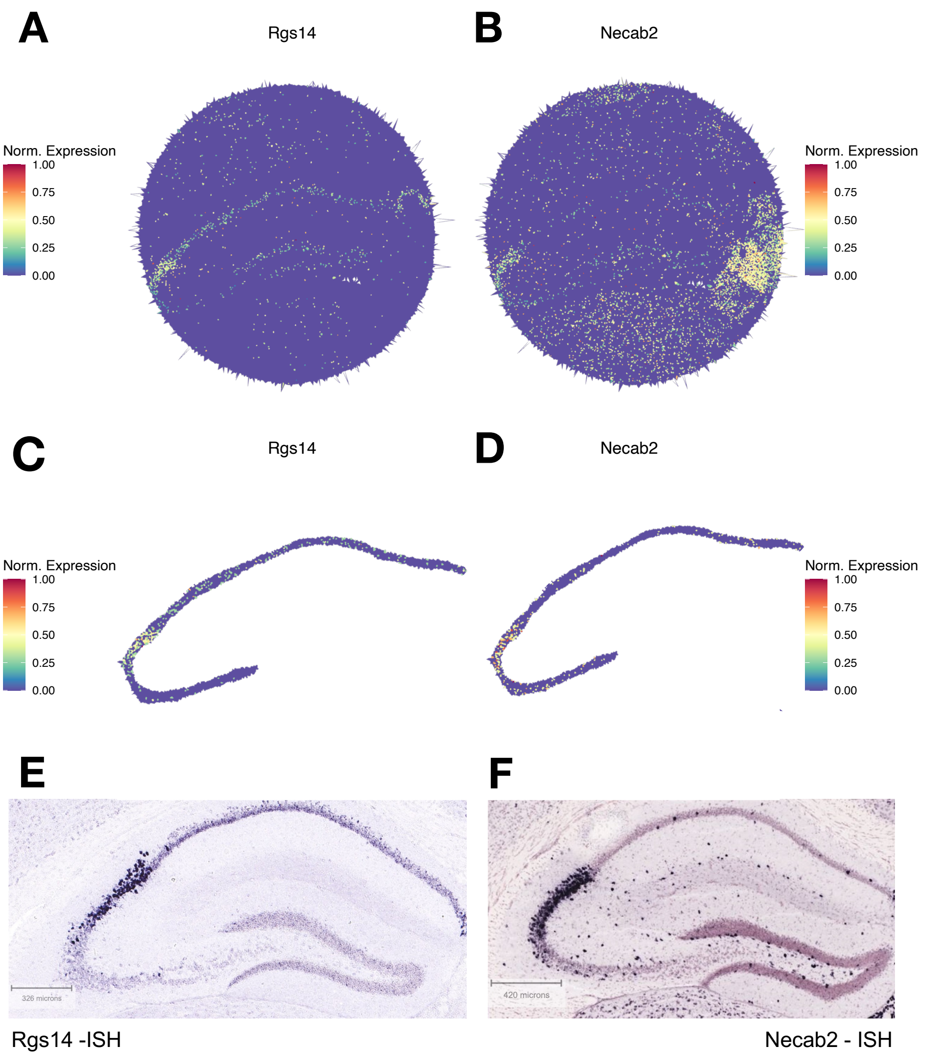

Appendix Figure S3 - Validation of CA2 field Marker genes.

A Vesalius shows that the overall expression of Rgs14 is weak throughout the entire but increase in the CA2 layer.

B Necab2 is only weakly expressed in the CA2 field.

C Rgs14 expression in the isolated CA field

D Necab2 expression in the isolated CA field

E In situ Hybridization Images taken from the Allen Brain Atlas for Rgs14 expression

F In situ Hybridization Images taken from the Allen Brain Atlas for Necab2 expression
