# Supplementary material for: Vesalius: high‐resolution in silico anatomization of spatial transcriptomic data using image analysis
Source: Mol Syst Biol. 2022 Sep 6;18(9):e11080. doi: 10.15252/msb.202211080 (PMC9446088; doi:10.15252/msb.202211080)
Supplement: Supplementary file 2 — EVFigs [file MSB-18-e11080-s006.pdf]

Expanded View Figures

Figure EV1. Vesalius methods visualized.

A Vesalius converts the transcriptome of cells by using a normalized latent space (either UMAP or PCA) and using embedding values as RGB color values.
B After image segmentation, Vesalius further isolates territories by pooling barcodes that are close to each other in 2D space. Vesalius finds all barcodes that are within a capture distance of each other (represented by red circle) and assigns a unique territory to all these barcodes. The same process is applied to all beads of that color segment until all beads have been pooled into a distinct spatial territory.
C Simulation regimes used to benchmark Vesalius in high-resolution ST data. The pure regime only contains one cell type per territory. The uniform regime contains n different cell types in equal proportion in each territory. The exponential regime contains n cell types in varying proportions between territories. The cell types are the same between territories. The dotted regime is made of a background territory with five circular territories of random size. The number of cell types between each territory is also randomized. Circular territories may overlap.
D Territory layering uses images representation of territory to iteratively select the edge of a territory and assign a layer value to that edge. Once all barcodes have been assigned to a layer, the number of layers can be reduced by merging neighboring layers.

© 2022 The Authors

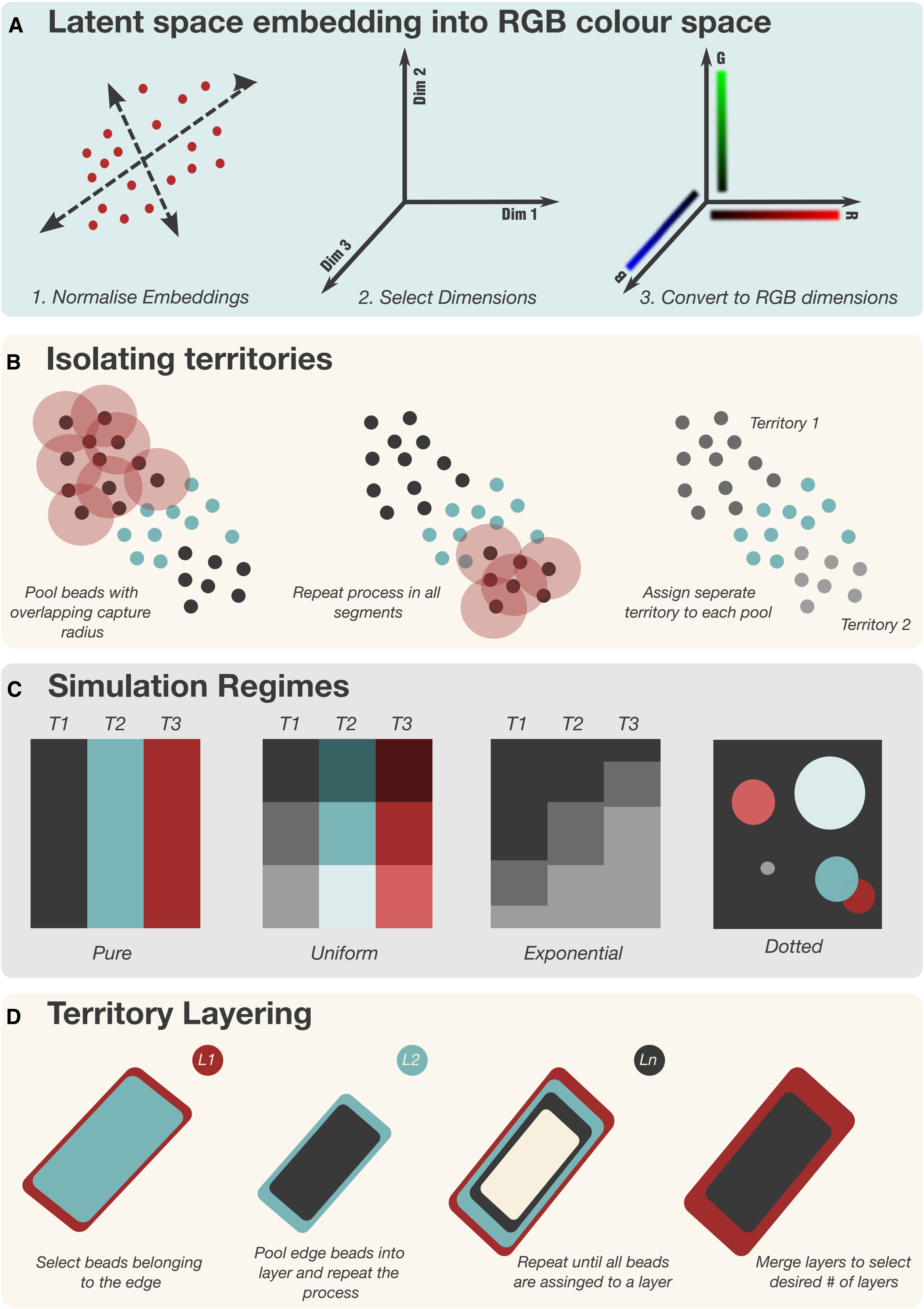

Figure EV1.

© 2022 The Authors

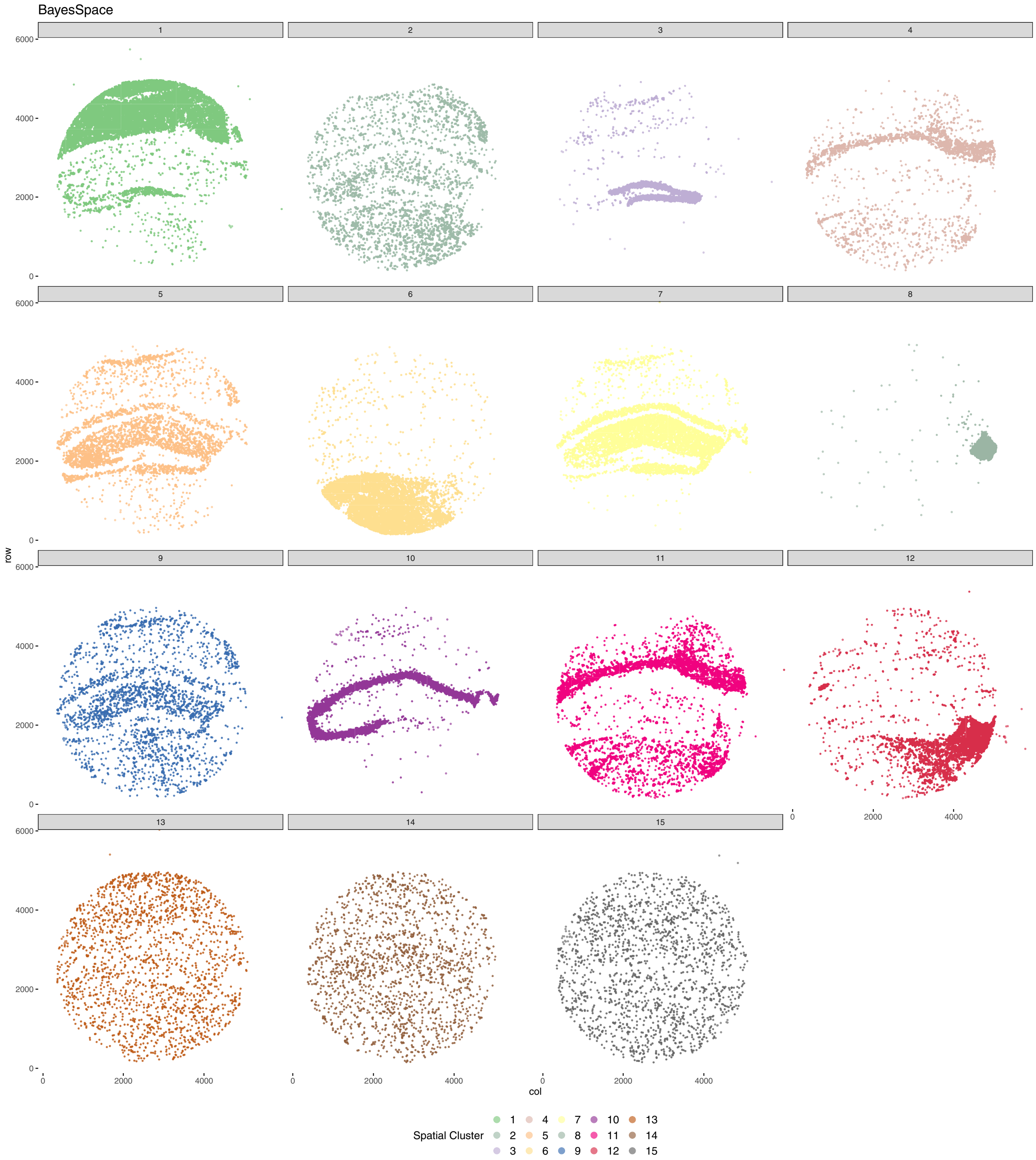

Figure EV2. BayesSpace spatial cluster in mouse hippocampus.

Visualizing BayesSpace clusters separately emphasizes that many of the spatial domains do not represent a specific anatomical structure. In this context, selecting a specific location in the tissue is impossible without either missing barcodes or by including unwanted barcodes. This also demonstrates why cluster sub-clustering does not equate to territory isolation and clustering.

© 2022 The Authors

Figure EV3. Seurat clusters in mouse hippocampus.

Visualizing Seurat clusters separately emphasizes that many of the clusters do not represent a specific anatomical structure. In this context, selecting a specific location in the tissue is impossible without either missing barcodes or by including unwanted barcodes. This also demonstrates why cluster sub-clustering does not equate to territory isolation and clustering. Seurat is not designed to recover spatial domains but rather cluster transcriptional similarity.

 © 2022 The Authors

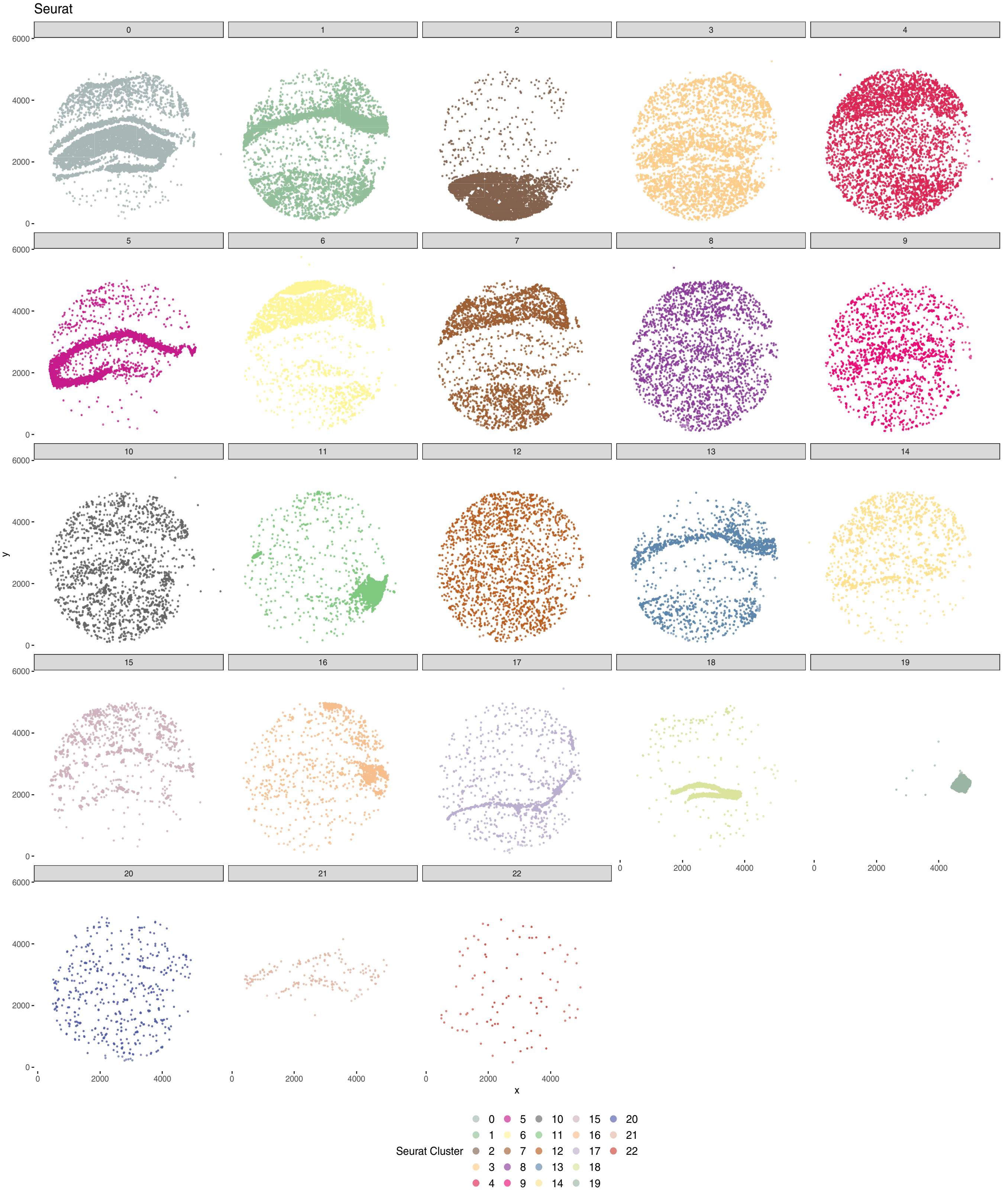
Seurat
0
1
2
3
4
5
6
7
8
9
10
11
12
13
14
15
16
17
18
19
20
21
22
6000
4000
2000
0
y
x
0
2000
4000
Seurat Cluster
0 1 2 3 4 5 6 7 8 9 10 11 12 13 14 15 16 17 18 19 20 21 22

Figure EV3.

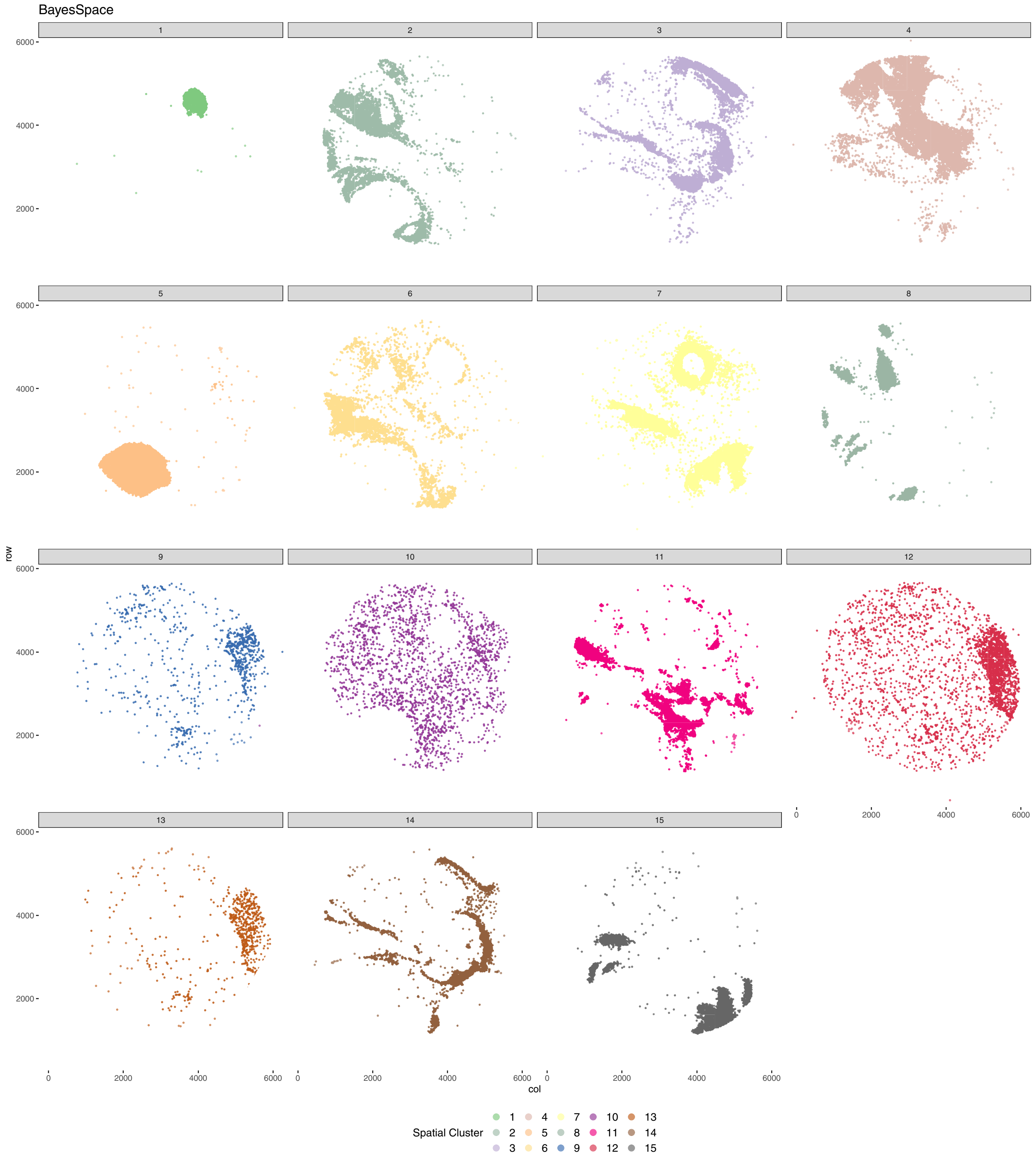

Figure EV4. BayesSpace spatial cluster in mouse embryo.

Visualizing BayesSpace clusters separately emphasizes that many of the spatial domains do not represent a specific anatomical structure. In this context, selecting a specific location in the tissue is impossible without either missing barcodes or by including unwanted barcodes. This also demonstrates why cluster sub-clustering does not equate to territory isolation and clustering.

© 2022 The Authors

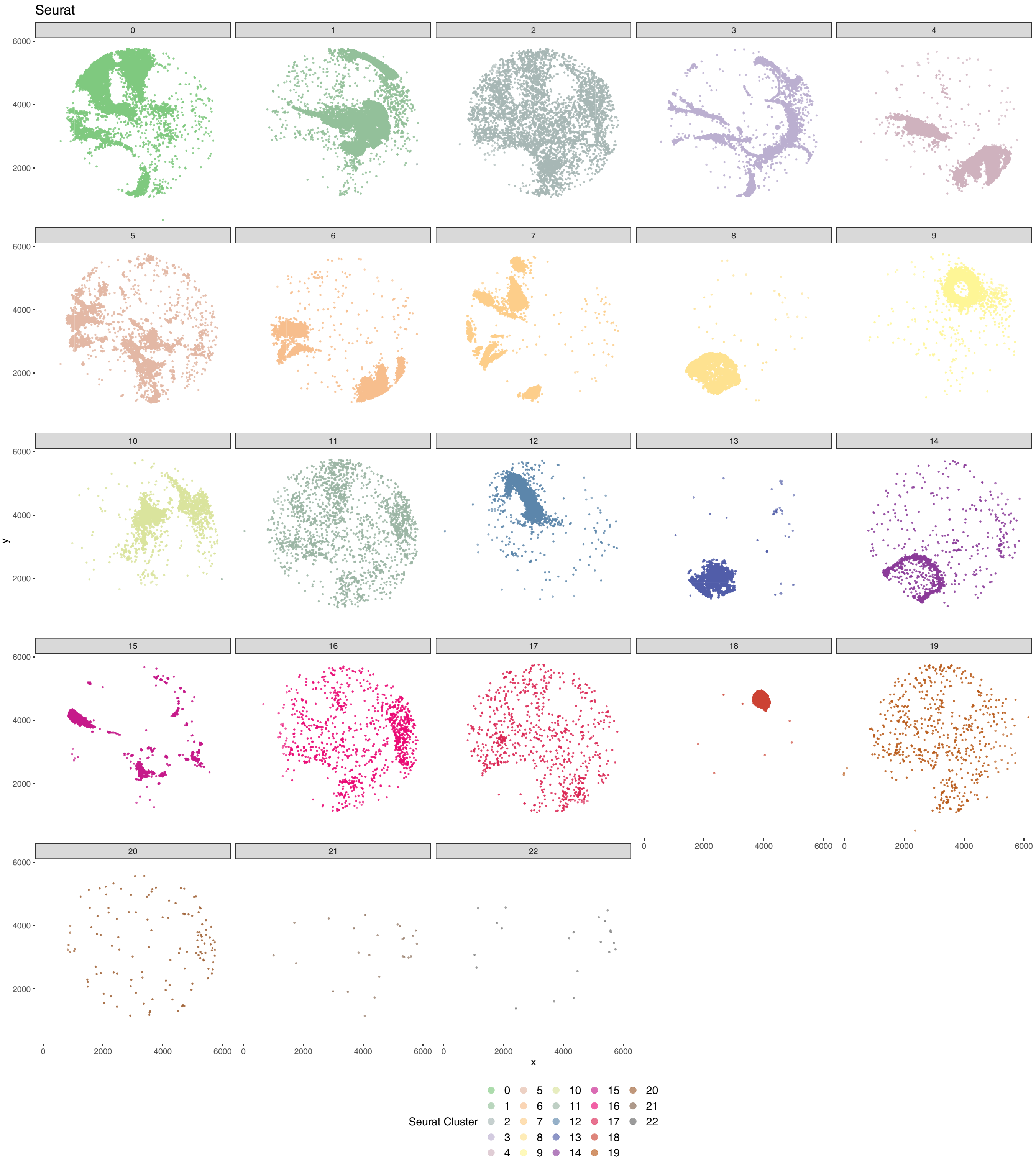

Figure EV5. Seurat clusters in mouse embryo.

Visualizing Seurat clusters separately emphasizes that many of the clusters do not represent a specific anatomical structure. In this context, selecting a specific location in the tissue is impossible without either missing barcodes or by including unwanted barcodes. This also demonstrates why cluster sub-clustering does not equate to territory isolation and clustering. Seurat is not designed to recover spatial domains but rather cluster transcriptional similarity.

© 2022 The Authors
