# Supplementary material for: Vesalius: high‐resolution in silico anatomization of spatial transcriptomic data using image analysis
Source: Mol Syst Biol. 2022 Sep 6;18(9):e11080. doi: 10.15252/msb.202211080 (PMC9446088; doi:10.15252/msb.202211080)
Supplement: Supplementary file 7 [file MSB-18-e11080-s007.pdf]

molecular systems biology

Vesalius: high-resolution anatomization of Spatial Transcriptomic data using Image Analysis.

Patrick Martin, Hyobin Kim, Cecilia Lövkvist, Byung-Woo Hong, and Kyoung Won
DOI: 10.15252/msb.202211080

Corresponding author(s): Kyoung Won (kyoungjae.won@cshs.org)

Review Timeline:

Submission Date:	18th Apr 22
Editorial Decision:	19th May 22
Revision Received:	8th Jul 22
Editorial Decision:	4th Aug 22
Revision Received:	18th Aug 22
Accepted:	19th Aug 22

Editor: Maria Polychronidou

Transaction Report:

(Note: With the exception of the correction of typographical or spelling errors that could be a source of ambiguity, letters and reports are not edited. Depending on transfer agreements, referee reports obtained elsewhere may or may not be included in this compilation. Referee reports are anonymous unless the Referee chooses to sign their reports.)

1st Editorial Decision **19th May 2022**

RE: MSB-2022-11080, Vesalius: high-resolution anatomization of Spatial Transcriptomic data using Image Analysis.

Thank you again for submitting your work to Molecular Systems Biology. We have now heard back from the three reviewers who agreed to evaluate your study. As you will see below, the reviewers think that the presented approach seems potentially interesting. However, they raise a series of concerns, which we would ask you to address in a major revision.

Without repeating all the comments listed below, some of the more fundamental issues are the following:

- Vesalius should be compared to more methods designed for spatial domain identification.

- The scalability and broad applicability of Vesalius should be better supported by additional analyses.

- The reviewers also list several technical concerns (including the need to present more technical details in the manuscript itself), which should be carefully addressed.

All issues raised by the referees would need to be satisfactorily addressed. Please let me know in case you would like to discuss in further detail any of the issues raised, I would be happy to schedule a call.

On a more editorial level, we would ask you to address the following points:

Reviewer #1:

In this manuscript, Martin et al. developed Vesalius to perform in silico tissue anatomization on high-resolution ST data by leveraging image processing techniques. The authors applied Vesalius on Slide-seq data from mouse brain and embryo, high resolution Seq-scope data from liver and colon, and proved Vesalius's ability to recover uniform territories. The authors then demonstrated that Vesalius could identify tissue territories with heterogenous cell compositions using synthetic ST data. Based on the identified territories, they further uncovered potential tissue sub-compartments with specific gene expression.

Overall, the study focused on an important question in the analysis of ST data -- spatial domain identification. It is interesting to embed transcriptome data into RGB color space. But the reviewer is concerned about the limited applicability of Vesalius. On one hand, the anatomization by Vesalius relied on the smoothing of the RGB-converted data, which can remove potential noise, but might lead to the missing of small tissue patches in actual conditions. On the other hand, Vesalius was neither applied on the FISH-based ST technologies, nor the low-resolution but more popular 10X Visium data. I have the following suggestions.

Major comments:

1. As the authors mentioned, Seurat was developed to perform clustering according to transcriptional similarity rather than to discover spatial domains. Vesalius should be compared with more methods which were specifically developed for spatial domain identification, such as stLearn, SpaGCN, STAGATE and so on.

2. The authors generated simulated ST data with 3 territories, and demonstrated that Vesalius could clearly distinguish territories under three different regimes. However, the case simulated above is the simplest one, in which clusters are linearly separable. How well will Vesalius perform in the anatomization of tissues with complex organization? I recommend the authors to simulate additional ST data with more complex data structure to prove the scalability of Vesalius.

3. I noticed that there were some isolated territories and NA territories in the examples of slide-seq V2 and Seq-scope, respectively. Could the authors comment on the isolated or NA territories? Should they be treated as the sequencing noise and ignored for downstream analysis, or they are biologically meaningful compartments?

4. As shown in Fig S2, both UMAP projection and PCA loading seems a little noisy. But k-means based on RGB-converted UMAP projection revealed coherent clustering result. I wonder whether the key is the regularization and smoothing of ST images. The smoothing of images can remove some noise to some extent. But on the other side, I'm concerned whether the smoothing would lead to the missing of some small patches with totally different cell types, which were captured by Seurat instead. By the way, more figures should be provided to visualize the results of intermediate steps for better interpretation of the smoothing process.

5. According to the case study of Slide-seq V2 and Stereo-seq, Vesalius seemed to be designed to identify spatial domains only in high-resolution sequencing-based ST data. Considering the assumption that spatial domains are coherent either in gene expression or cell-type compositions still holds true in FISH-based technologies or low-resolution ST techniques, could the authors demonstrate Vesalius's compatibility with different ST platforms?

Minor comments:

1. Vesalius requires the user to choose k value for K-means clustering. In many cases, it is hard to choose appropriate k especially when there's no prior knowledge. Is it possible for Vesalius to determine k automatically according to some metrics, such as Silhouette score?

2. The orientation of mouse hippocampus in the main figures (Fig. 2, 3, 4) is inconsistent with that in supplementary figures (Fig. S2, S3), and so is the case of mouse embryo. Please adjust the figures accordingly.

3. When visualizing the clustering result of different methods, please make sure the colors of the corresponding domains across different methods are as consistent as possible for convenient and intuitive comparing. For example, in Fig 2b, the color palette made the clustering result of Seurat look more undistinguishable than the actual.

4. The figure legends should be more detailed. For example, in Fig 2f, do the points in the box plots represent multiple simulation replicates?

5. In Fig 3c, the title of the color bar should be 'Pcp4 Exp.', rather than 'Pcp Exp.'.

6. There are some grammar mistakes in the article especially in the part of method. Please check the syntax throughout the manuscript.

Reviewer #2:

This manuscript provides a new approach to segmenting tissues with high-resolution ST data. I fully agree with the importance of identifying spatial domains. Thus, I expect this paper will suggest another cell metadata (i.e. territory) for ST data analysis. Because of not enough depth and cellular heterogeneity of spatial tile, as needed, we need to consider tile(cell)-territory instance to find DEGs between same cell types on different territories. Furthermore, because each tile of low-resolution bears transcriptomic profiles from multiple cells, we may handle the data as bulk-RNA seq or pseudo bulk RNA-seq with tissue territory information.

This paper has faithfully investigated and answered the current issues of ST assay. 1) low-resolution ST assay may contain heterogenous cell types on a tile. 2) high-resolution ST assay may assign different barcodes to different subcellular organelles of a single cell. 3) Lack of noise-reducing approach for ST image. At least for these three main issues, the authors have obviously investigated and confirmed that Vesalius is working well.

However, in order to publish this paper and to be a common tool for tissue territory identification, there are some points as below:

Major
1. [Normalization strategy] Before dimensionality reduction for scRNA-seq analysis, in general, we normalize and perform the dimensional reduction. Although some papers show the result that log normalization is the best way for normalizing scRNA-seq data, some other normalization approaches are also useful. Likewise, in order to assess PCA, selecting features is the most important for PCA results. Thus, I wonder which normalization approaches and features for PCA are optimal for Vesalius. (at least scater and scran R package)
2. [Informative PCs] It is a good idea that the authors adopt RGB based image analyzers such as imager. The authors show the results from two modes such as UMAP and PCA mode. These modes commonly use PCA. Then, how did the authors choose informative PCs? Using Elbowplot? On the other hand, when we use 3D-UMAP, because there are only three coordinates for each tile, the embedding step is fit to RGB values. But in PCA mode, we must have more than three informative PCs. Although the results look different in Fig. S2, the result with PC13-PC15 looks useful. Can we consider another image analyzer with more dimensions for the input vector? And please let me know the differences between the results in Fig.S2 according to different PC sets. Finally, please add a function to report informative PCs.
3. [Variable territory boundary thresholds] In the Seurat pipeline, when we do the clustering analysis, we can choose 'resolution' to generate a proper number of clusters. Likewise, can Vesalius show a different number of territories? I think you can make variants by changing the number of neighbors and minimal distance when you do 3D-UMAP.
4. [Heterogeneity]: The authors show the performance of Vesalius to recover territories with simulation. As they mentioned they selected barcodes assigned with only one cell type after performing cell type deconvolution. How many cells with multiple cell types? As I know Slide-seq V2 is high-resolution (subcellular resolution), then is it possible that Vesalius combines subcellular organelles to single cell?
Minor
5. Please add statistical significances for simulation data (Fig. 2f and 2g)
6. In order to take higher citation, you should mention more application points on the discussion part such as that Vesalius helps comparison between same cell types on different territories. Because Vesalius can suggest a novel layer for cell(tile) metadata to analyze ST dataset.
7. Please revise typos and mis-annotations (Fig XX & Fig. XX)
8. Please use the same colors for at least Vesalius on Fig. 2e

Reviewer #3:

In their manuscript "Vesalius: high-resolution in silico anatomization of Spatial Transcriptomic data using Image Analysis." Martin et al. propose a novel computational method "Vesalius" implemented in R and provided as a package together with online documentation. Vesalius identifies regions that supposedly correspond to anatomical structures based on high-resolution spatial transcriptomics (ST) data such as slide-seq. The authors compare and benchmark their new method against two published tools (BayesSpace and Giotto) that had been created for a similar purpose in lower resolution ST data and one tool (Seurat) the purpose of which is to place cells in space but not identify regions. Martin et al show that their tool works well in simulated and real data and in several examples, it is demonstrated how biological insight can be generated using Vesalius-based analysis. The presented tool adds nicely to the existing toolbox that allows the community to leverage and interpret spatial transcriptomics data, especially supporting high-resolution ST methods. Finally, I would like to highlight the innovative approach of representing the expression data as RGB encoded images. Apart from the good results that the authors present so far using "conventional"

image analysis methods (e.g. smoothing and color-clustering) this in principle also allows the employment of more powerful machine-learning based tools from the computer vision field that have been specifically developed for image segmentation.
The manuscript is well written and the data and concepts are nicely displayed. I do not have any major concerns.
My minor concerns/suggestions are the following:

1. As much as I find the approach of RGB encoding innovative and full of potential, I have doubts that this "fancy hack" is really necessary for the presented purpose. Rather, it seems that similar results could be achieved by simply reducing the resolution of high-resolution data by binning and smoothing spatially proximal measurements in expression space (not RGB image space) and then continuing with existing tools. If the RGB embedding is really crucial, this should be made more clear and supported by additional analysis (e.g. contrasting to clustering in binned/smoothed expression space) if applicable.

2. The term "anatomization" for the process of territory assignment performed by Vesalius is a bit misleading because it implies the recovery of pre-defined anatomical structures, that sometimes might have more historical rather than biological basis. Anatomical structures that are determined by their cell type composition will most likely be correctly identified but others that are rather characterized for example by their anatomical position, won't. The direct comparison in Figure 2a illustrates this, where some but by far not all anatomical annotations find their corresponding territories. So, what Vesalius in fact does is a biologically and functionally meaningful tissue segmentation that can but not must correspond to known anatomical structures.

MSB-2022-11080 - Vesalius: high-resolution anatomization of Spatial Transcriptomic data using Image Analysis.

Thanks a lot for reconstructive reviews. Please find our point-to-point responses. For clarity, we provide our response to reviewer comments in a color-coded manner. Blue represents the reviewer's concern, in black our response (if any) and in red the changes we made to address the comments. We also include the text changes and the newly generated figures (when appropriate). The text changes within the manuscript itself are highlighted in red.

Reviewer #1:

In this manuscript, Martin et al. developed Vesalius to perform in silico tissue anatomization on high-resolution ST data by leveraging image processing techniques. The authors applied Vesalius on Slide-seq data from mouse brain and embryo, high resolution Seq-scope data from liver and colon, and proved Vesalius's ability to recover uniform territories. The authors then demonstrated that Vesalius could identify tissue territories with heterogenous cell compositions using synthetic ST data. Based on the identified territories, they further uncovered potential tissue sub-compartments with specific gene expression.

Overall, the study focused on an important question in the analysis of ST data -- spatial domain identification. It is interesting to embed transcriptome data into RGB color space. But the reviewer is concerned about the limited applicability of Vesalius. On one hand, the anatomization by Vesalius relied on the smoothing of the RGB-converted data, which can remove potential noise, but might lead to the missing of small tissue patches in actual conditions. On the other hand, Vesalius was neither applied on the FISH-based ST technologies, nor the low-resolution but more popular 10X Visium data. I have the following suggestions.

Major comments:

1. As the authors mentioned, Seurat was developed to perform clustering according to transcriptional similarity rather than to discover spatial domains. Vesalius should be compared with more methods which were specifically developed for spatial domain identification, such as stLearn, SpaGCN, STAGATE and so on.

We thank the reviewer for this suggestion. To provide a more in-depth comparison between Vesalius and other spatial domain identification methods, we added SEDR, SpaGCN and STAGATE in addition of BayesSpace and Giotto to our benchmarking in simulated data sets. We compared both the ability to recover territories and run time. The results of the benchmarking are found in Figure 2.

Page 6 – Section: Vesalius outperforms other Spatial domains tools in heterogenous territories
Page 21 – Section: Method Comparison

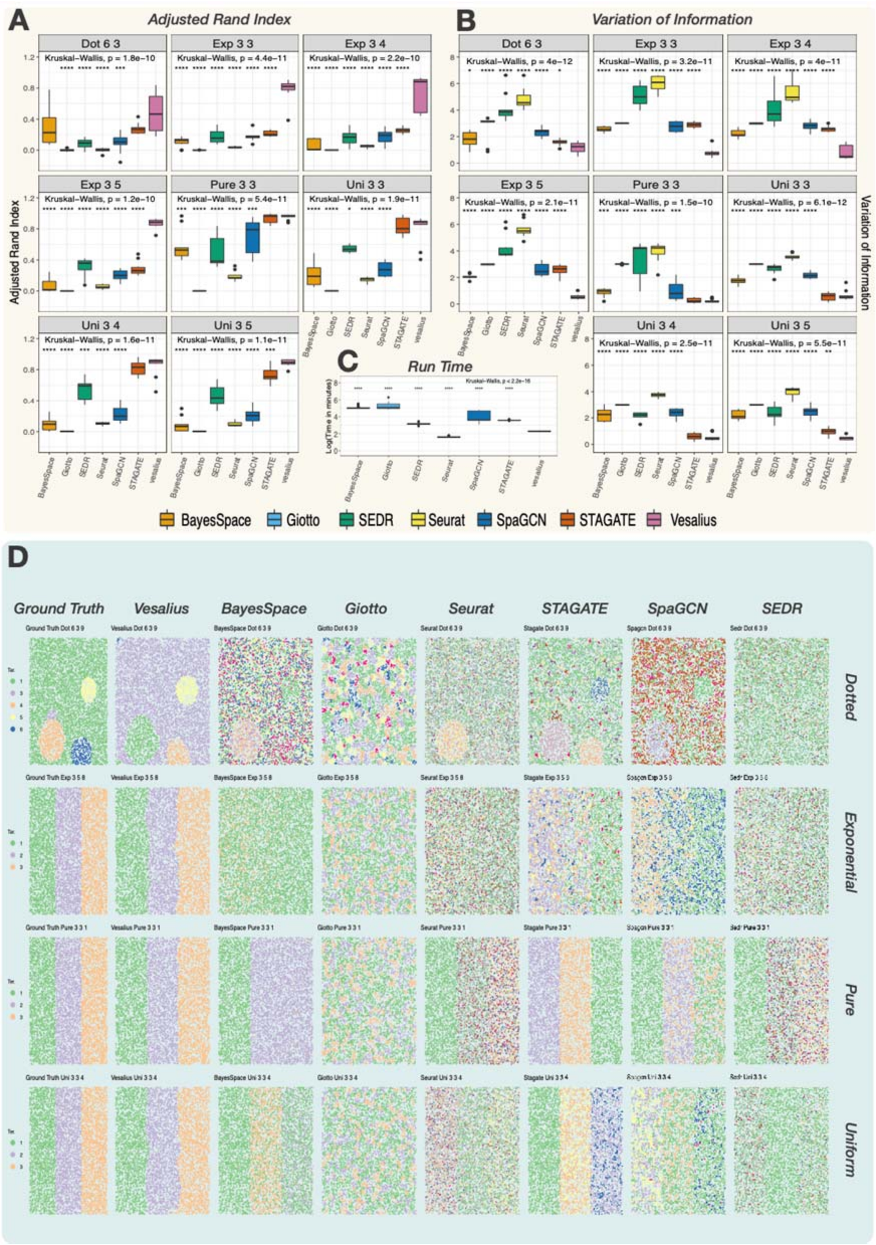

Figure 2 - Vesalius outperforms other Spatial domains tools in heterogenous territories.

A Adjusted Rand Index score between Vesalius, BayesSpace, Giotto, Seurat, STAGATE, SpaGCN and SEDR. Overall, Vesalius outperforms other tools in retrieving spatial domains in high resolution simulated data sets (Kruskal-Wallis

and star notation using Wilcoxon Rank Sum test for the multiple comparison with n = 10 simulation replicates).

B The high performance of Vesalius compared to competitors is also highlighted using the Variation of Information score which is more robust against cluster granularity. Boxplot show VI scores over 10 simulation replicates. (Kruskal-Wallis and star notation using Wilcoxon Rank Sum test for the multiple comparison with n = 10 simulation replicates)

C Vesalius has the lowest run time amongst spatial domain tools with a run time 3 to 20 times lower than competing tools. (Kruskal-Wallis and star notation using Wilcoxon Rank Sum test for the multiple comparison with n = 80 simulation replicates)

D Example of simulated ground truth and prediction by each tool in four simulated regimes (Dotted, Exponential, Pure and Uniform). The label above each plot describes the simulation run in the following format Tool – Regime – Number of territories – Number of cells – Simulation replicate. For example, Vesalius Uni 3 3 1 describes Vesalius's prediction in the uniform regime which contains 3 territories each with 4 cell types in replicate 1.

2. The authors generated simulated ST data with 3 territories, and demonstrated that Vesalius could clearly distinguish territories under three different regimes. However, the case simulated above is the simplest one, in which clusters are linearly separable. How well will Vesalius perform in the anatomization of tissues

with complex organization? I recommend the authors to simulate additional ST data with more complex data structure to prove the scalability of Vesalius.

In addition to our three earlier simulated ST data sets, we created another regime named dotted. The dotted regime contains a background territory in which 5 circular territories are placed. The radius of each territory is randomly selected, and the center of each territory is placed upon a randomly selected coordinate pair. Each territory including the background contains a random number of cell types (between 1 to 3 cell types) and for each cell type we randomly sample barcodes. The circular territories may overlap with each other providing a more complex organization of tissue territories. A description of the simulation regimes is shown in Figure EV1C. The results of the benchmarking including the dotted regime are shown in Figure 2 (Please see the answer 1).
Page 6 – Section: Vesalius outperforms other Spatial domains tools in heterogenous territories
Page 21 – Section: Simulating high resolution ST data and tissue heterogeneity

3. I noticed that there were some isolated territories and NA territories in the examples of slide-seq V2 and Seq-scope, respectively. Could the authors comment on the isolated or NA territories? Should they be treated as the sequencing noise and ignored for downstream analysis, or they are biologically meaningful compartments?

We thank the reviewer for noticing this. The NA's was in fact an oversight due to a minor bug when plotting the figures. We have corrected the plots. As for the isolated territories, these territories represent territories that contain too little barcodes and that are too far away from another territory within the same color cluster. The minimum number of barcodes and the capture radius (Described in the methods and illustrated in Figure EV1B) are user defined parameters. As such, it is possible for no territories to be considered "isolated" and each of these territories can be investigated in greater detail through territory isolation.

However, for clarity purposes in our figures, we decided to use more stringent parameters and isolate the small patches under the isolated territory label. The biologically validity of these compartments will only become clear after careful investigation and will be dependent on the data set.

We removed NA's as it no longer exists

Page 19 – Section: Vesalius – Isolating Territories

"Territories may be assigned to the isolated group. This group is defined by territories that did not contain enough cells (user defined parameter) and outside of the capture radius (user defined). "

4. As shown in Fig S2, both UMAP projection and PCA loading seems a little noisy. But k-means based on RGB-converted UMAP projection revealed coherent clustering result. I wonder whether the key is the regularization and smoothing of ST images. The smoothing of images can remove some noise to some extent. But on the other side, I'm concerned whether the smoothing would lead to the missing of some small patches with totally different cell types, which were captured by Seurat instead. By the way, more figures should be provided to visualize the results of intermediate steps for better interpretation of the smoothing process.

I agree with the reviewer that smoothing helps to remove noise, but it may come at the cost of losing smaller patches. We can see this in the simulation results under the dotted regime where Vesalius merged a smaller territory into one of the larger ones (Figure 2D). It occurred in the dotted regime with high complexity and a large amount of noise. In fact, all other tools we tested struggled to distinguish ground truth territories and failed to handle this heterogeneity. It is still of note that Vesalius provided the most accurate representation of ground truth in all regimes.

Furthermore, in Figure 4, we demonstrate that the isolation of uniform territories increases the level of detail that we can recover from ST data. For example, the

isolation and in-depth analysis of the CA field, the medial habenula and the embryonic eye showed structures beyond what Seurat could achieve on its own. While the smoothing may initially lose some of the finer details, these details and much more can easily be recovered in downstream analysis. With Vesalius, we aim to demonstrate that uniform territories are an asset to understanding tissue heterogeneity.

To illustrate the effects of image processing on ST data, we added Appendix Figure S2 that shows raw Vesalius images, Vesalius images after regularization, smoothing, segmentation and finally after territory isolation. We provide these examples in both Slide-seq V2 data sets used in the manuscript (mouse hippocampus and mouse embryo).

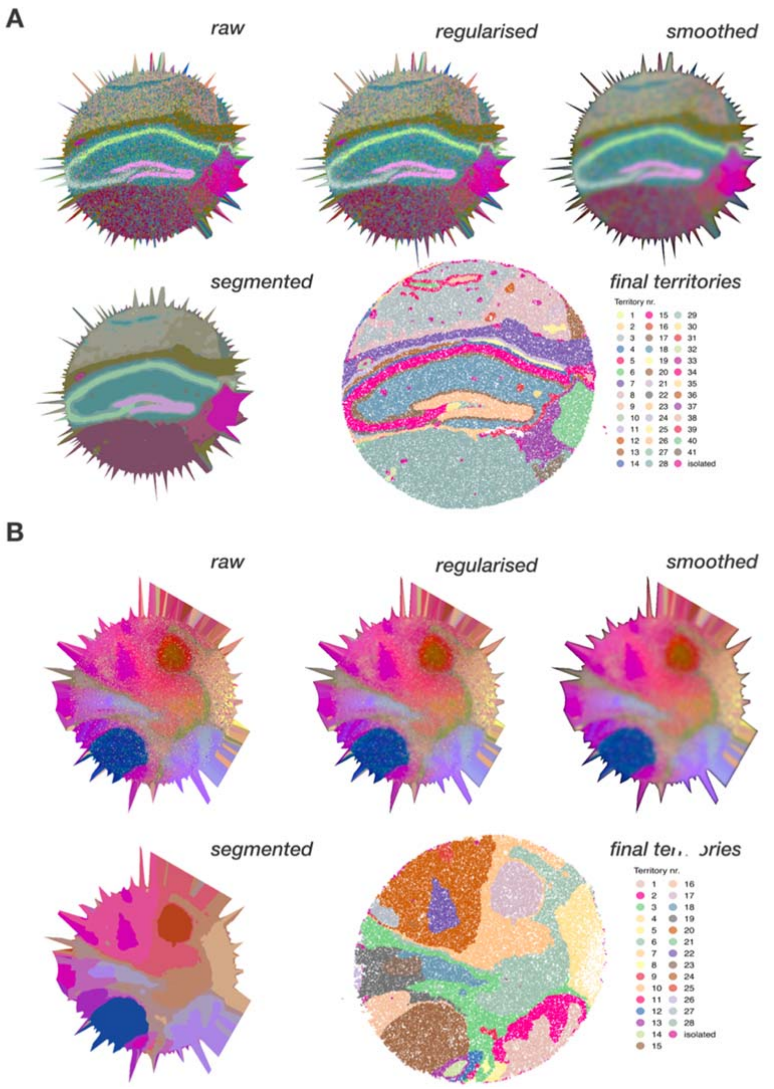

Appendix Figure S2. Demonstrating the effects of image processing on Vesalius images.

5. According to the case study of Slide-seq V2 and Stereo-seq, Vesalius seemed to be designed to identify spatial domains only in high-resolution sequencing-based ST data. Considering the assumption that spatial domains are coherent either in gene expression or cell-type compositions still holds true in FISH-based technologies or low-resolution ST techniques, could the authors demonstrate Vesalius's compatibility with different ST platforms?

To demonstrate the broad applicability of Vesalius, we tested Vesalius in seqFISH mouse embryo (Fig. 3G) as well as 3 Visium 10X data sets including mouse brain, mouse kidney and human breast cancer (Fig 3H-J). We believe that our results demonstrate that our method is applicable to any ST method. Vesalius can recover tissue territories (including different brain layers) in seqFISH despite the lower number of probed genes. In lower resolution Visium 10X data sets, Vesalius recovers clear territories in a wide variety of tissues.

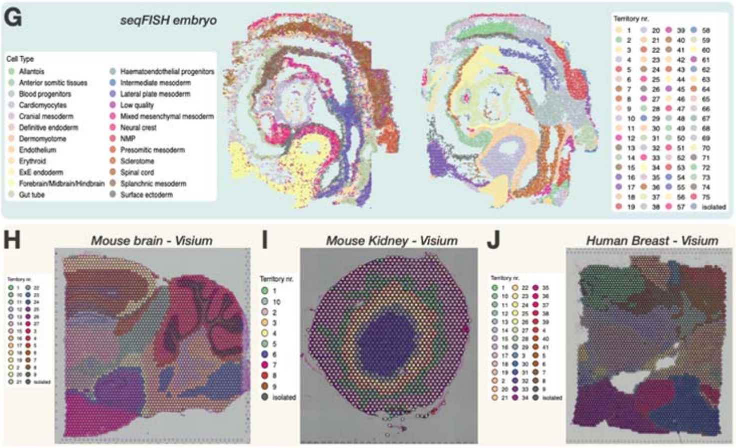

Figure 3 G - Vesalius recovers tissue territories in seqFISH data in mouse embryo (right) and can distinguish between different brain regions compared to the single cell annotation (left).

H,I,J In low resolution Visium 10X data sets, Vesalius accurately recover tissue territories in a broad range of tissue such as mouse brain (H), mouse kidney (**I**), and human breast cancer (**J**).

Page 8-9 – Section: Vesalius illustrates tissue territories in a broad range of ST data sets

Minor comments:

1. Vesalius requires the user to choose k value for K-means clustering. In many cases, it is hard to choose appropriate k especially when there's no prior knowledge. Is it possible for Vesalius to determine k automatically according to some metrics, such as Silhouette score?

At the moment, the value of *k* must be selected by the user. However, in the case of Vesalius, the value of *k* serves a similar purpose as granularity resolution in Louvain or Leiden clustering. *K* only defines the number of colors that should be extracted from the RGB color space (color depth). These colors are then separated into independent territories. This can be visualized in Supplementary Figure S3 between the segmented images and the final territories. Modulating the value of *k* allows the user to select and modulate the granularity of the territories.

Page 18-19 – Section: Vesalius – Image processing and segmentation

"While the number of color clusters is fixed by k (or final k) as described above, the final number of territories may vary depending on parameter selection used throughout the analysis. This includes images processing and territory isolation. The selection of these parameters depends on the users interests and how they wish to explore ST data. In the context, the value of k serves a similar role as the granularity resolution in Louvain or Leiden clustering."

2. The orientation of mouse hippocampus in the main figures (Fig. 2, 3, 4) is inconsistent with that in supplementary figures (Fig. S2, S3), and so is the case of mouse embryo. Please adjust the figures accordingly.
We have adjusted the orientation of the figures.

3. When visualizing the clustering result of different methods, please make sure the colors of the corresponding domains across different methods are as consistent as possible for convenient and intuitive comparing. For example, in Fig 2b, the color palette made the clustering result of Seurat look more undistinguishable than the actual.
We agree with the reviewer that the choice of color palette is important to visualize clustering results. We faced the same difficulty when finding an appropriate color palette for Vesalius. The main issue we face is that there are no good color palettes when the number of groups exceeds 20. It becomes extremely difficult to distinguish colors that are similar to each other. After trying over a dozen different palettes, we found that randomizing colors from a palette reduces the chance of similar colors being next to each other. We set a seed to ensure reproducibility but due to differences in number of clusters the resulting palette after randomization will differ. While this is not an ideal situation, we do not know of a method to generate clear and consistent color palette for so many groups.

We randomized the color palette used for Seurat in Figure 3B to increase the clarity of the clustering results.

4. The figure legends should be more detailed. For example, in Fig 2f, do the points in the box plots represent multiple simulation replicates?
Each box plot shows performance over the 10 simulated replicates.

We have added more detail to the figure legends to increase clarity.

5. In Fig 3c, the title of the color bar should be 'Pcp4 Exp.', rather than 'Pcp Exp.'.

We have amended the figure.

6. There are some grammar mistakes in the article especially in the part of method. Please check the syntax throughout the manuscript.

We have reviewed our article and hopefully have eliminated all syntax mistakes.

Reviewer #2:

This manuscript provides a new approach to segmenting tissues with high-resolution ST data. I fully agree with the importance of identifying spatial domains. Thus, I expect this paper will suggest another cell metadata (i.e. territory) for ST data analysis. Because of not enough depth and cellular heterogeneity of spatial tile, as needed, we need to consider tile(cell)-territory instance to find DEGs between same cell types on different territories. Furthermore, because each tile of low-resolution bears transcriptomic profiles from multiple cells, we may handle the data as bulk-RNA seq or pseudo bulk RNA-seq with tissue territory information.

This paper has faithfully investigated and answered the current issues of ST assay. 1) low-resolution ST assay may contain heterogenous cell types on a tile. 2) high-resolution ST assay may assign different barcodes to different subcellular organelles of a single cell. 3) Lack of noise-reducing approach for ST image. At least for these three main issues, the authors have obviously investigated and confirmed that Vesalius is working well.

However, in order to publish this paper and to be a common tool for tissue territory identification, there are some points as below:

Major

1. [Normalization strategy] Before dimensionality reduction for scRNA-seq

analysis, in general, we normalize and perform the dimensional reduction. Although some papers show the result that log normalization is the best way for normalizing scRNA-seq data, some other normalization approaches are also useful. Likewise, in order to assess PCA, selecting features is the most important for PCA results. Thus, I wonder which normalization approaches and features for PCA are optimal for Vesalius. (at least scater and scran R package)

We agree that data pre-processing is a key element of scRNA-seq analysis. As described in the methods, all the pre-processing prior to generating Vesalius images is carried out by Seurat regardless of the data type (Visium, SlideSeq V2, SeqScope, and SeqFISH). This includes data normalization (log normalization) and features selection (top 2000 variable features). Besides Seurat, log normalization has been used in several studies using spatial transcriptomics including Lohoff et al (Nature Biotechnology, 2022) and Mantri et al. (PMID: 33741943). We agree with the reviewer that there is an ongoing debate on which normalization method is the most suitable for scRNAseq and spatial transcriptomics data. We selected log normalization to remain consistent in our comparison with other tools as they all use log normalization.

We have clarified these points in text.

Page 16 – Section: Pre-processing

"Pre-processing of data prior to Vesalius image building was handled by the Seurat package(Satija et al, 2015)*. Data sets were loaded, log normalized and scaled using the default Seurat settings. Variable features (n=2000) were extracted prior to PCA. Log Normalization was preferred for pre-processing as it would provide a fair comparison to other tools and methods. Selecting the number of Principle Components used for analysis can be achieved by using the Seurat JackStraw function or the Seurat ElbowPlot function."*

2. [Informative PCs] It is a good idea that the authors adopt RGB based image analyzers such as imager. The authors show the results from two modes such as UMAP and PCA mode. These modes commonly use PCA. Then, how did the authors choose informative PCs? Using Elbowplot? On the other hand, when we

use 3D-UMAP, because there are only three coordinates for each tile, the embedding step is fit to RGB values. But in PCA mode, we must have more than three informative PCs. Although the results look different in Fig. S2, the result with PC13-PC15 looks useful. Can we consider another image analyzer with more dimensions for the input vector? And please let me know the differences between the results in Fig.S2 according to different PC sets. Finally, please add a function to report informative PCs.

We thank the reviewer for this insightful comment. In our case, we used the default 30 PCs provided by Seurat. Selecting the number of Principle Components used for analysis can be done by using the Seurat JackStraw function or the Seurat ElbowPlot function. If this is of interest, we suggest using these functions prior to generating Vesalius images.

With the exception of Appendix Figure S1 (previously Figure S2), all territory plots are made using the default UMAP mode. We recommend using this mode as UMAP provides a straightforward way of representing the data in 3 dimensions. Effectively, we reduced 30 PCs to a 3-dimensional UMAP projection. PCA mode is only provided as a way of visualizing PCA slices. PC slices (3 PCs in each slice – PCs used are shown above each plot) are shown in Appendix Figure S1 alongside the territories that would result from using PC slices.

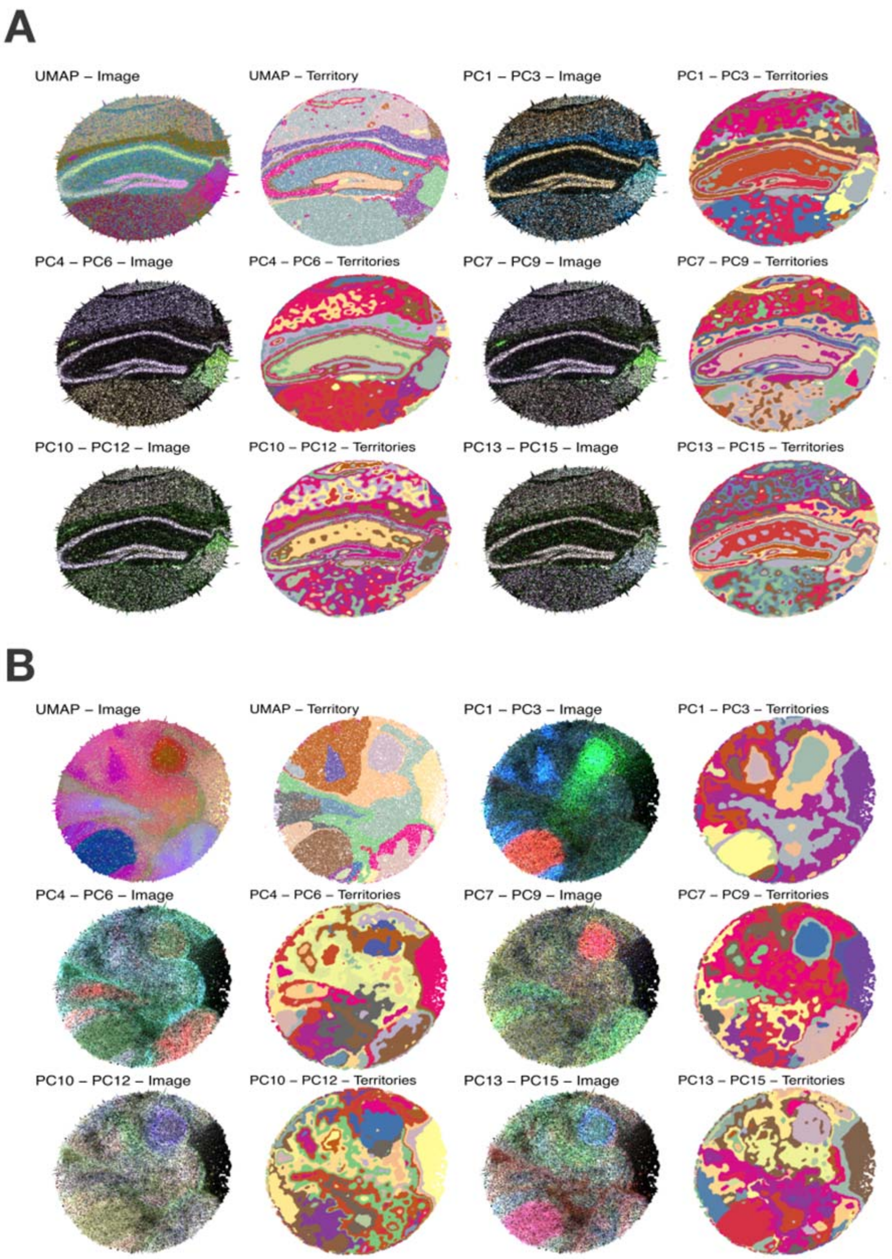

Appendix Figure S1 - UMAP projection and PCA loading value define similar yet not identical territories

We have clarified these points in text.

Page 16 – Section: Pre-processing

"Selecting the number of Principle Components used for analysis can be achieved by using the Seurat JackStraw function or the Seurat ElbowPlot function. "

3. [Variable territory boundary thresholds] In the Seurat pipeline, when we do the clustering analysis, we can choose 'resolution' to generate a proper number of clusters. Likewise, can Vesalius show a different number of territories? I think you can make variants by changing the number of neighbors and minimal distance when you do 3D-UMAP.

This is an excellent observation from the reviewer. In the case of Vesalius, the number of territories is defined by the image processing and territory isolation parameters. By varying these parameters, Vesalius can show many different territory combinations that can reflect the specific interest of the user. This can vary from a broad view of tissue territories to a fine grain perspective. This can be visualized in Appendix Figure S3.
Page 18-19 – Section: Vesalius – Image processing and segmentation

"While the number of color clusters is fixed by k (or final k) as described above, the final number of territories may vary depending on parameter selection used throughout the analysis. This includes images processing and territory isolation. The selection of these parameters depends on the users interests and how they wish to explore ST data. In the context, the value of k serves a similar role as the granularity resolution in Louvain or Leiden clustering."

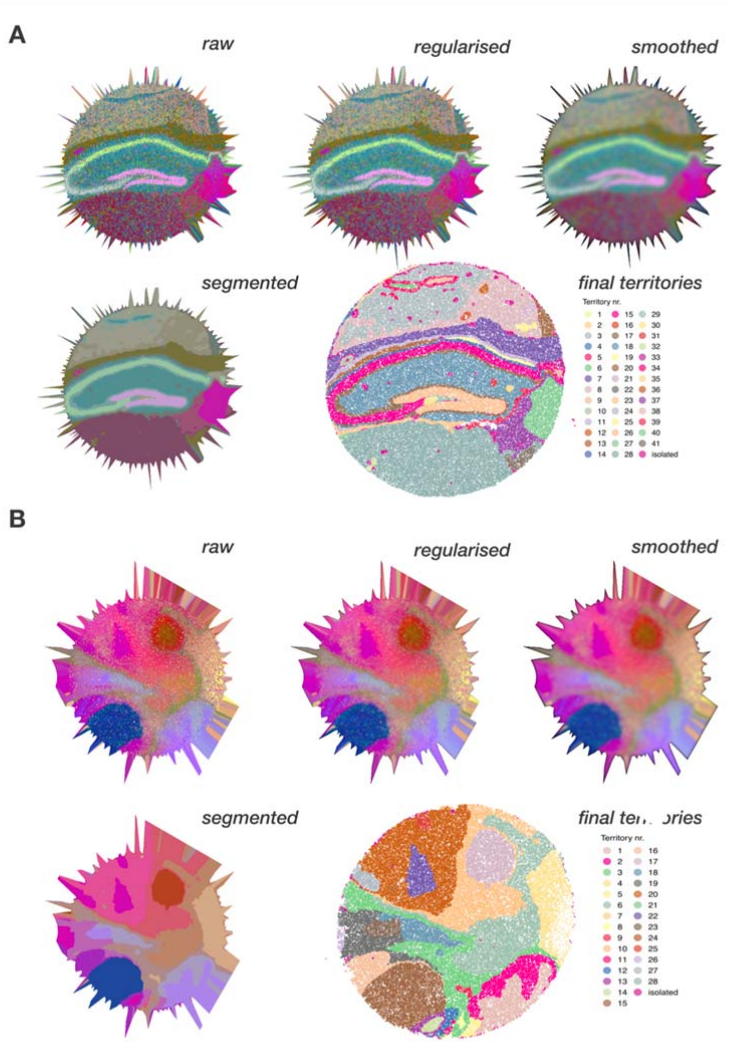

Appendix Figure S2 - Demonstrating the effects of image processing on Vesalius images.

4. [Heterogeneity]: The authors show the performance of Vesalius to recover territories with simulation. As they mentioned they selected barcodes assigned with only one cell type after performing cell type deconvolution. How many cells with multiple cell types? As I know Slide-seq V2 is high-resolution (subcellular resolution), then is it possible that Vesalius combines subcellular organelles to single cell?

We selected barcodes containing a single cell type to simulate biological complexity more accurately. We did not include barcodes containing multiple cell

types as this would be reflective of technical complexity. Slide-seqV2 in mouse hippocampus contains over 53 000 unique barcodes. After some filtering to only include cell types with at least 50 unique barcodes, we ended up with 11 957 barcodes across 13 cell types taken from mouse hippocampus data. In sub-cellular resolution data, Vesalius will indeed combine subcellular organelles into an overall territory that then can be investigate in further detail through downstream analysis. However, it should be noted that the resolution of Slide-seq V2 is around 10 micro-meters which we believe is too low to accurately study subcellular organelle expression patterns.

Page 16 – Section: Cell type deconvolution
Page 20 – Section: Simulating high resolution ST data and tissue heterogeneity

"First, we applied RCTD(Cable et al, 2021) to deconvolute cell types and selected barcodes that contained a single cell type (n = 12 013). From these barcodes, we selected cell types that contained at least 50 different barcodes leaving 11 957 barcodes across 13 cell types."

Minor
5. Please add statistical significances for simulation data (Fig. 2f and 2g)
We added statistical significance for the simulation data. We used an Kruskal-Wallis test for overall performance and a Wilcoxon Rank Sum test for multiple comparison. Significance is shown using exact p value and star notation.
Page 21 – Section: Method comparison

"Statistical analysis was performed to compared overall performance between methods over 10 simulation replicates. We first tested for normality using a Shapiro Wilks test, then we tested the homogeneity of variance with a Bartlett test using base R functionality. We compared all groups with a Kruskal-Wallis test and used a Wilcoxon Rank Sum test for multiple comparison. P values for the Kruskal-Wallis are shown in the figure (Fig 2A-C) and multiple comparison is

shown using start notation for clarity. Comparisons were carried out using the ggpubr R package. "

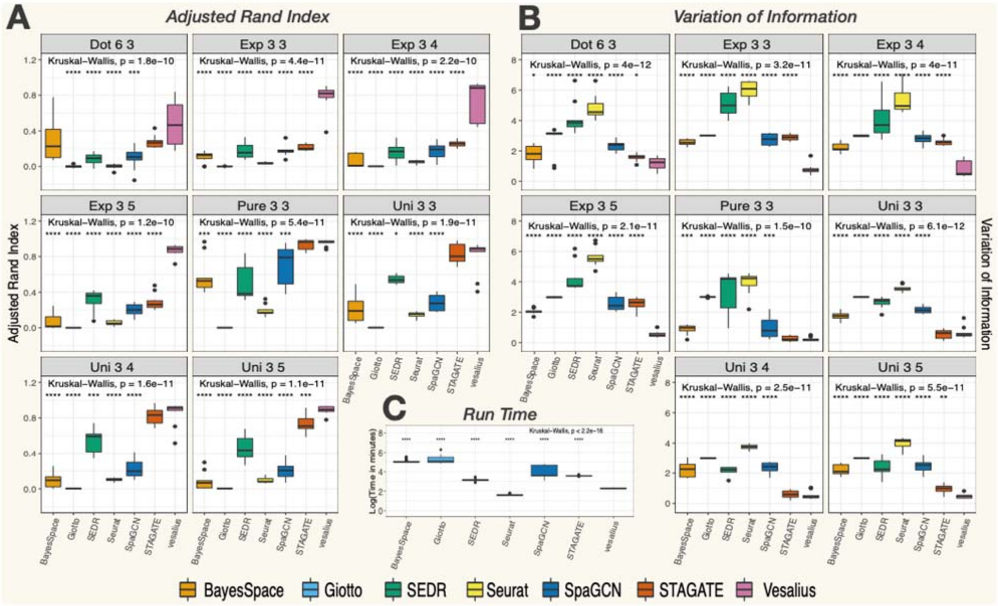

Figure 2 A-C

6. In order to take higher citation, you should mention more application points on the discussion part such as that Vesalius helps comparison between same cell types on different territories. Because Vesalius can suggest a novel layer for cell(tile) metadata to analyze ST dataset.

We thank the reviewer for this excellent suggestion. We have added this suggestion in text.

Page 15 – Section: Discussion

"Vesalius complements other ST analysis methods, enhances potential biological insights, and may help in providing an additional layer to cell type annotation based on their spatial location. "

7. Please revise typos and mis-annotations (Fig XX & Fig. XX)

We have made the change to the text to fix these mis-annotations.

8. Please use the same colors for at least Vesalius on Fig. 2e

We have changed the colors to remain as consistent as possible between ground truth as other tools including Vesalius.

Reviewer #3:

In their manuscript "Vesalius: high-resolution in silico anatomization of Spatial Transcriptomic data using Image Analysis." Martin et al. propose a novel computational method "Vesalius" implemented in R and provided as a package together with online documentation. Vesalius identifies regions that supposedly correspond to anatomical structures based on high-resolution spatial transcriptomics (ST) data such as slide-seq. The authors compare and benchmark their new method against two published tools (BayesSpace and Giotto) that had been created for a similar purpose in lower resolution ST data and one tool (Seurat) the purpose of which is to place cells in space but not identify regions. Martin et al show that their tool works well in simulated and real data and in several examples, it is demonstrated how biological insight can be generated using Vesalius-based analysis. The presented tool adds nicely to the existing toolbox that allows the community to leverage and interpret spatial transcriptomics data, especially supporting high-resolution ST methods. Finally, I would like to highlight the innovative approach of representing the expression data as RGB encoded images. Apart from the good results that the authors present so far using "conventional" image analysis methods (e.g. smoothing and color-clustering) this in principle also allows the employment of more powerful machine-learning based tools from the computer vision field that have been specifically developed for image segmentation.

The manuscript is well written and the data and concepts are nicely displayed. I do not have any major concerns.

We thank the review for his interest in our method and recognizing its potential.

My minor concerns/suggestions are the following:

1. As much as I find the approach of RGB encoding innovative and full of potential, I have doubts that this "fancy hack" is really necessary for the presented purpose. Rather, it seems that similar results could be achieved by simply reducing the resolution of high-resolution data by binning and smoothing spatially proximal measurements in expression space (not RGB image space) and then continuing with existing tools. If the RGB embedding is really crucial, this should be made more clear and supported by additional analysis (e.g. contrasting to clustering in binned/smoothed expression space) if applicable.

Clustering using binned/smoothed expression in a higher dimensional space could be considered. If 3-dimensional UMAPs are used to represent expression space, this will be equivalent to the RGB code that we used.

By incorporating RGB colour code, Vesalius is extremely fast (Figure 2) and utilise all functions developed for image processing. Also, the strength of Vesalius resides more in the representation of the transcriptome into an image array format. This image representation means that we do not need to sacrifice resolution in order to smooth spatially proximal barcodes as the smoothing occurs over pixels and not barcodes.

2. The term "anatomization" for the process of territory assignment performed by Vesalius is a bit misleading because it implies the recovery of pre-defined anatomical structures, that sometimes might have more historical rather than biological basis. Anatomical structures that are determined by their cell type composition will most likely be correctly identified but others that are rather characterized for example by their anatomical position, won't. The direct comparison in Figure 2a illustrates this, where some but by far not all anatomical annotations find their corresponding territories. So, what Vesalius in fact does is a biologically and functionally meaningful tissue segmentation that can but not must correspond to known anatomical structures.

We examined the entire manuscript and replaced “anatomization” into “territory detection” or appropriate words when the distinction helps to the best of our ability.

For example:

Page 7 – Section: Vesalius overcomes the challenge of isolating tissue territories containing heterogenous cell populations**.**

RE: MSB-2022-11080R, Vesalius: high-resolution anatomization of Spatial Transcriptomic data using Image Analysis.

Thank you for sending us your revised manuscript. We have now heard back from the two reviewers who were asked to evaluate your revised study. As you will see below, both reviewers are satisfied with the performed revisions and are supportive of publication. Reviewer #1 only lists a couple of minor issues, which we would ask you to fix in a final round of revision. We would also ask you to address a few minor editorial issues listed below.

Reviewer #1:

The authors have addressed my issues and I have no major issues with the study. Several minor issues related to the figures.

Fig 2

1. As the authors described in the figure legend, the first number in the label above each plot of Fig 2D represents the number of territories. However, the number '6' is inconsistent with '5' territories in the example of the dotted regime (i.e., the first row in Fig 2D). The same applies to all dotted-regime-related plots in Fig 2.
2. The description in the legend of Fig 2D, 'For example, Vesalius Uni 3 3 1 describes Vesalius's prediction in the uniform regime which contains 3 territories each with 4 cell types in replicate 1' should be changed into 'For example, Vesalius Uni 3 3 1 describes Vesalius's prediction in the uniform regime which contains 3 territories each with 3 cell types in replicate 1'.

Fig 3
It seems that the shape of the selected region in the right plot (Seurat) from Fig 3B has been changed. Please adjust it to make it consistent with the shape in the left panel.

Reviewer #2:

The authors have addressed all my concerns, and I am happy that the revised manuscript is ready for publication!

MSB-2022-11080 - Vesalius: high-resolution anatomization of Spatial Transcriptomic data using Image Analysis.

Thanks a lot for reconstructive reviews. Please find our point-to-point responses. For clarity, we provide our response to reviewer comments in a color-coded manner. Blue represents the reviewer's concern, in black our response (if any) and in red the changes we made to address the comments. We also include the text changes and the newly generated figures (when appropriate). The text changes within the manuscript itself are highlighted in red.

Reviewer #1:

The authors have addressed my issues and I have no major issues with the study. Several minor issues related to the figures.

Fig 2
1. As the authors described in the figure legend, the first number in the label above each plot of Fig 2D represents the number of territories. However, the number '6' is inconsistent with '5' territories in the example of the dotted regime (i.e., the first row in Fig 2D). The same applies to all dotted-regime-related plots in Fig 2.

We adjusted the labels to reflect the composition of the ground truth. We also clarify the explanation for the dotted regime to reflect the difference in territory numbers.

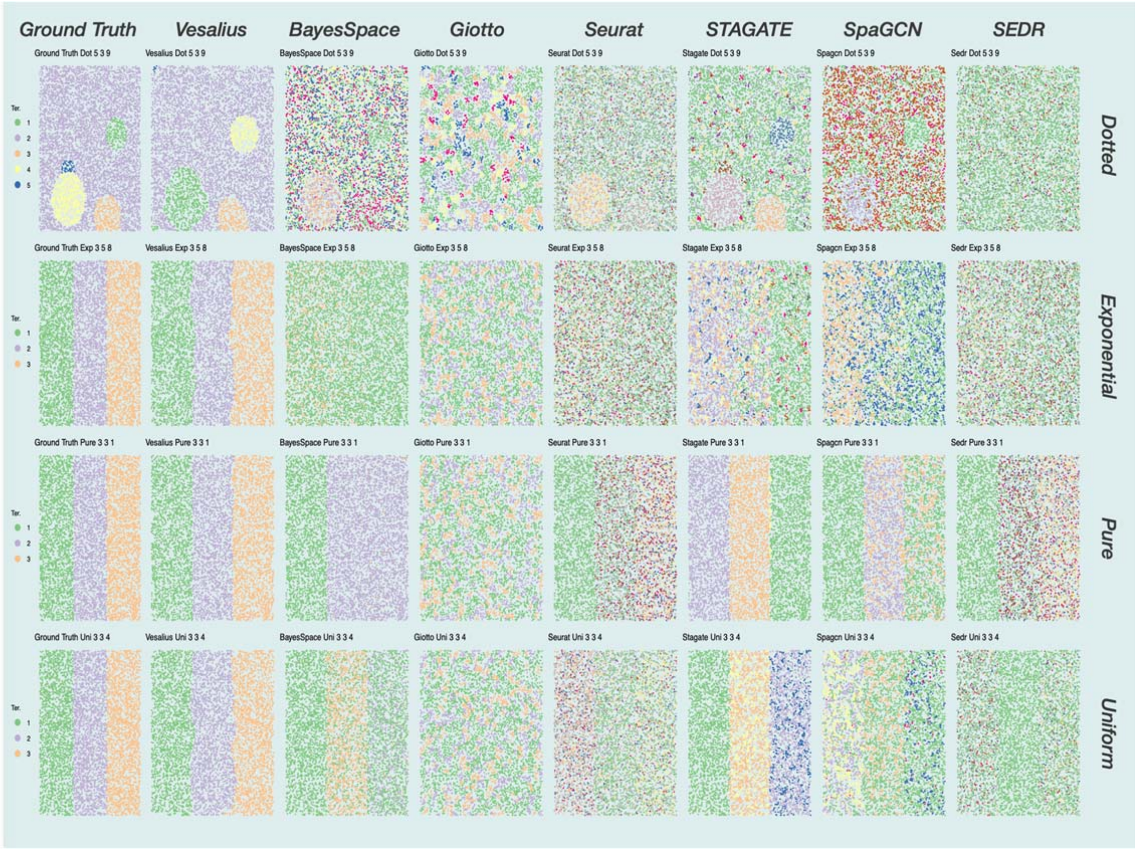

Methods – Page 20 - Simulating high resolution ST data and tissue heterogeneity

"To further increase the complexity of the dotted regime, we also allow for overlaps between territories. While the input parameters to our simulation should produce 6 total territories, allowing overlaps means that some territories may completely mask another."

2. The description in the legend of Fig 2D, 'For example, Vesalius Uni 3 3 1 describes Vesalius's prediction in the uniform regime which contains 3 territories each with 4 cell types in replicate 1' should be changed into 'For example, Vesalius Uni 3 3 1 describes Vesalius's prediction in the uniform regime which contains 3 territories each with 3 cell types in replicate 1'.

We have made changes to the figure caption as suggested.

Figure 2

"For example, Vesalius Uni 3 3 1 describes Vesalius's prediction in the uniform regime which contains 3 territories each with 3 cell types in replicate 1. "

Fig 3
It seems that the shape of the selected region in the right plot (Seurat) from Fig 3B has been changed. Please adjust it to make it consistent with the shape in the left panel.

We have adjusted the figure.

Reviewer #2:

The authors have addressed all my concerns, and I am happy that the revised manuscript is ready for publication!

2nd Revision - Editorial Decision 19th Aug 2022

Manuscript number: MSB-2022-11080RR
Title: Vesalius: high-resolution anatomization of Spatial Transcriptomic data using Image Analysis.

Thank you again for sending us your revised manuscript. We are now satisfied with the modifications made and I am pleased to inform you that your paper has been accepted for publication.

EMBO Press Author Checklist

Corresponding Author Name: Arjun Raj
Journal Submitted to: Molecular Systems Biology
Manuscript Number: MSB-2022-10979

USEFUL LINKS FOR COMPLETING THIS FORM
The EMBO Journal - Author Guidelines
EMBO Reports - Author Guidelines
Molecular Systems Biology - Author Guidelines
EMBO Molecular Medicine - Author Guidelines

Reporting Checklist for Life Science Articles (updated January
This checklist is adapted from Materials Design Analysis Reporting (MDAR) Checklist for Authors. MDAR establishes a minimum set of requirements in transparent reporting in the life sciences (see Statement of Task: 10.31222/osf.io/9sm4x). Please follow the journal's guidelines in preparing your
Please note that a copy of this checklist will be published alongside your article.

Abridged guidelines for figures
1. Data
The data shown in figures should satisfy the following conditions:
- the data were obtained and processed according to the field's best practice and are presented to reflect the results of the experiments in an accurate and unbiased manner.
- ideally, figure panels should include only measurements that are directly comparable to each other and obtained with the same assay.
- plots include clearly labeled error bars for independent experiments and sample sizes. Unless justified, error bars should not be shown for technical
- if n<5, the individual data points from each experiment should be plotted. Any statistical test employed should be justified.
- Source Data should be included to report the data underlying figures according to the guidelines set out in the authorship guidelines on Data

2. Captions
Each figure caption should contain the following information, for each panel where they are relevant:
- a specification of the experimental system investigated (eg cell line, species name).
- the assay(s) and method(s) used to carry out the reported observations and measurements.
- an explicit mention of the biological and chemical entity(ies) that are being measured.
- an explicit mention of the biological and chemical entity(ies) that are altered/varied/perturbed in a controlled manner.
- the exact sample size (n) for each experimental group/condition, given as a number, not a range;
- a description of the sample collection allowing the reader to understand whether the samples represent technical or biological replicates (including how many animals, litters, cultures, etc.).
- a statement of how many times the experiment shown was independently replicated in the laboratory.
- definitions of statistical methods and measures:
  - common tests, such as t-test (please specify whether paired vs. unpaired), simple χ2 tests, Wilcoxon and Mann-Whitney tests, can be unambiguously identified by name only, but more complex techniques should be described in the methods section;
  - are tests one-sided or two-sided?
  - are there adjustments for multiple comparisons?
  - exact statistical test results, e.g., P values = x but not P values < x;
  - definition of 'center values' as median or average;
  - definition of error bars as s.d. or s.e.m.

Please complete ALL of the questions below.
Select "Not Applicable" only when the requested information is not relevant for your study.

Materials

**Newly Created Materials**	**Information included in the manuscript?**	**In which section is the information available?** (Reagents and Tools Table, Materials and Methods, Figures, Data Availability Section)
New materials and reagents need to be available; do any restrictions apply?	Not Applicable	

**Antibodies**	**Information included in the manuscript?**	**In which section is the information available?** (Reagents and Tools Table, Materials and Methods, Figures, Data Availability Section)
For **antibodies** provide the following information:<br>- Commercial antibodies: RRID (if possible) or supplier name, catalogue number and or/clone number<br>- Non-commercial: RRID or citation	Not Applicable	

**DNA and RNA sequences**	**Information included in the manuscript?**	**In which section is the information available?** (Reagents and Tools Table, Materials and Methods, Figures, Data Availability Section)
**Short novel DNA or RNA including primers, probes**: provide the sequences.	Not Applicable	

**Cell materials**	**Information included in the manuscript?**	**In which section is the information available?** (Reagents and Tools Table, Materials and Methods, Figures, Data Availability Section)
**Cell lines:** Provide species information, strain. Provide accession number in repository **OR** supplier name, catalog number, clone number, and/**OR** RRID.	Not Applicable	
**Primary cultures:** Provide species, strain, sex of origin, genetic modification status.	Not Applicable	
Report if the cell lines were recently **authenticated** (e.g., by STR profiling) and tested for mycoplasma contamination.	Not Applicable	

**Experimental animals**	**Information included in the manuscript?**	**In which section is the information available?** (Reagents and Tools Table, Materials and Methods, Figures, Data Availability Section)
**Laboratory animals or Model organisms:** Provide species, strain, sex, age, genetic modification status. Provide accession number in repository **OR** supplier name, catalog number, clone number, **OR** RRID.	Not Applicable	
**Animal observed in or captured from the field:** Provide species, sex, and age where possible**.**	Not Applicable	
Please detail **housing and husbandry conditions**.	Not Applicable	

**Plants and microbes**	**Information included in the manuscript?**	**In which section is the information available?** (Reagents and Tools Table, Materials and Methods, Figures, Data Availability Section)
**Plants:** provide species and strain, ecotype and cultivar where relevant, unique accession number if available, and source (including location for collected wild specimens).	Not Applicable	
**Microbes:** provide species and strain, unique accession number if available, and source.	Not Applicable	

**Human research participants**	**Information included in the manuscript?**	**In which section is the information available?** (Reagents and Tools Table, Materials and Methods, Figures, Data Availability Section)
If collected and within the bounds of privacy constraints report on age, sex and gender or ethnicity for all study participants.	Not Applicable	

**Core facilities**	**Information included in the manuscript?**	**In which section is the information available?** (Reagents and Tools Table, Materials and Methods, Figures, Data Availability Section)
If your work benefited from core facilities, was their service mentioned in the acknowledgments section?	Not Applicable	

Design

Study protocol	Information included in the manuscript?	In which section is the information available? (Reagents and Tools Table, Materials and Methods, Figures, Data Availability Section)
If study protocol has been **pre-registered, provide DOI in the manuscript**. For clinical trials, provide the trial registration number **OR** cite DOI.	Not Applicable	
Report the **clinical trial registration number** (at ClinicalTrials.gov or equivalent), where applicable.	Not Applicable	

Laboratory protocol	Information included in the manuscript?	In which section is the information available? (Reagents and Tools Table, Materials and Methods, Figures, Data Availability Section)
Provide DOI OR other citation details if **external detailed step-by-step protocols** are available.	Not Applicable	

Experimental study design and statistics	Information included in the manuscript?	In which section is the information available? (Reagents and Tools Table, Materials and Methods, Figures, Data Availability Section)
Include a statement about **sample size** estimate even if no statistical methods were used.	Not Applicable	
Were any steps taken to minimize the effects of subjective bias when allocating animals/samples to treatment (e.g. **randomization procedure**)? If yes, have they been described?	Not Applicable	
Include a statement about **blinding** even if no blinding was done.	Not Applicable	
Describe **inclusion/exclusion criteria** if samples or animals were excluded from the analysis. Were the criteria pre-established?<br><br>If sample or data points were omitted from analysis, report if this was due to attrition or intentional exclusion and provide justification.	Not Applicable	
For every figure, are **statistical tests** justified as appropriate? Do the data meet the assumptions of the tests (e.g., normal distribution)? Describe any methods used to assess it. Is there an estimate of variation within each group of data? Is the variance similar between the groups that are being statistically compared?	Yes	Figures, Materials and Methods

Sample definition and in-laboratory replication	Information included in the manuscript?	In which section is the information available? (Reagents and Tools Table, Materials and Methods, Figures, Data Availability Section)
In the figure legends: state number of times the experiment was **replicated** in laboratory.	Yes	Figures
In the figure legends: define whether data describe **technical or biological replicates**.	Yes	Figures

Ethics

Ethics	Information included in the manuscript?	In which section is the information available? (Reagents and Tools Table, Materials and Methods, Figures, Data Availability Section)
Studies involving **human participants**: State details of **authority granting ethics approval** (IRB or equivalent committee(s), provide reference number for approval.	Not Applicable	
Studies involving **human participants**: Include a statement confirming that **informed consent** was obtained from all subjects and that the experiments conformed to the principles set out in the WMA Declaration of Helsinki and the Department of Health and Human Services Belmont Report.	Not Applicable	
Studies involving **human participants:** For publication of **patient photos**, include a statement confirming that consent to publish was obtained.	Not Applicable	
Studies involving experimental **animals**: State details of **authority granting ethics approva**l (IRB or equivalent committee(s), provide reference number for approval. Include a statement of compliance with ethical regulations.	Not Applicable	
Studies involving **specimen and field samples:** State if relevant **permits** obtained, provide details of authority approving study; if none were required, explain why.	Not Applicable	

Dual Use Research of Concern (DURC)	Information included in the manuscript?	In which section is the information available? (Reagents and Tools Table, Materials and Methods, Figures, Data Availability Section)
Could your study fall under dual use research restrictions? Please check biosecurity documents and list of **select agents and toxins** (CDC): https://www.selectagents.gov/sat/list.htm	Not Applicable	
If you used a select agent, is the security level of the lab appropriate and reported in the manuscript?	Not Applicable	
If a study is subject to dual use research of concern regulations, is the name of the **authority granting approval and reference number** for the regulatory approval provided in the manuscript?	Not Applicable	

Reporting

The MDAR framework recommends adoption of discipline-specific guidelines, established and endorsed through community initiatives. Journals have their own policy about requiring specific guidelines and recommendations to complement MDAR.

Adherence to community standards	Information included in the manuscript?	In which section is the information available? (Reagents and Tools Table, Materials and Methods, Figures, Data Availability Section)
State if relevant guidelines or checklists (e.g., **ICMJE, MIBBI, ARRIVE, PRISMA**) have been followed or provided.	Not Applicable	
For **tumor marker prognostic studies,** we recommend that you follow the **REMARK** reporting guidelines (see link list at top right). See author guidelines, under 'Reporting Guidelines'. Please confirm you have followed these guidelines.	Not Applicable	
For **phase II and III randomized controlled trials**, please refer to the **CONSORT** flow diagram (see link list at top right) and submit the CONSORT checklist (see link list at top right) with your submission. See author guidelines, under 'Reporting Guidelines'. Please confirm you have submitted this list.	Not Applicable	

Data Availability

Data availability	Information included in the manuscript?	In which section is the information available? (Reagents and Tools Table, Materials and Methods, Figures, Data Availability Section)
Have **primary datasets** been deposited according to the journal's guidelines (see 'Data Deposition' section) and the respective accession numbers provided in the Data Availability Section?	Not Applicable	
Were **human clinical and genomic datasets** deposited in a public access-controlled repository in accordance to ethical obligations to the patients and to the applicable consent agreement?	Not Applicable	
Are **computational models** that are central and integral to a study available without restrictions in a machine-readable form? Were the relevant accession numbers or links provided?	Yes	Data availability
If publicly available data were reused, provide the respective **data citations in the reference list**.	Yes	References
